# Interactions of a medicinal climber *Tinospora cordifolia* with supportive interspecific plants trigger the modulation in its secondary metabolic profiles

**DOI:** 10.1038/s41598-019-50801-0

**Published:** 2019-10-04

**Authors:** Bhawana Sharma, Aarti Yadav, Rajesh Dabur

**Affiliations:** Department of Biochemistry, Maharshi Dayanand University, Rohtak, Haryana 124001 India

**Keywords:** Mass spectrometry, Chemical ecology

## Abstract

*Tinospora cordifolia* (*TC*) is scientifically proven immunomodulatory drug being used for centuries. Ancient literature reported that inter-specific interactions change medicinal properties of *TC*. Thus, the current study is aimed to understand the influence of interspecific biotic interactions on chemo-profiles of *TC*. To explore it, *TC* samples collected from six co-occurring plants, *i*.*e*. *Azarditchita indica*, *Acacia nilotica*, *Albezia lebbeck*, *Ficus benghalensis*, *Tamarandus indica* and *Acacia leucophloea* were analyzed by HPLC-ESI-QTOF-MS. Mass data were subjected to multivariate analysis. Support vector machines (SVMs) was found to be best classifier (r^2^ < 0.93). Data analysis showed the specific compounds in all *TC* due to inter-specific interactions. Data were further analyzed with SNK post-hoc test followed by permutative (n = 50) Bonferroni FDR multiple testing correction. The compound without any missing values reduced the number of variables to 133 (p < 0.01). Statistical analysis revealed that *TC* having interactions with *A*.*lebbeck* and *A*. *nilotica* formed the most distant groups. However, *TC* co-occurred with *A*. *indica* showed the highest number of up-regulated metabolites, including jatrorrhizine, chrysin, peonidin, 6-methylcoumarin and some terpenoids. Some metabolites, including jatrorrhizine and magnoflorine were quantified to confirm the accuracy of qualitative analysis. Results demonstrated the influence of inter-specific biotic interactions on *TC* chemo-profiles, hence its medicinal properties.

## Introduction

*Tinospora cordifolia* (Willd.) Miers, a climbing shrub of the family Menispermaceae, is a well known Ayurvedic medicinal herb with different names including *rasayana* (to purify the blood), *amrita* (to bring the dead back to life), nectar of immortality and heavenly elixir. *Ayurveda* and Chinese traditional medicinal systems describe the use of *T*. *cordifolia* not only as a health tonic but also for the treatment of a large number of diseases including diabetes, asthma, liver and platelet damages, stress and cancer^1^. The plant has been reported for its diverse pharmacological properties, *i*.*e*. immunomodulatory, hepatoprotective, neuroprotective and nephroprotective^2–6^. *T*. *cordifolia* that grows in co-occurrence with *Azaditchia*. *indica* and *Magnifera indica* is the best for medicinal efficacy in Ayurveda. Recently, it has been shown that *T*. *cordifolia* showed best immunomodulatory activity on interaction with *A*. *indica*^7,8^. Our lab has documents that phytochemical constituents (*i*.*e*. tinosporaside) were significantly higher when *T*. *cordifolia* co-occured with *A*. *indica*^9^. These studies indicate that the medicinal properties of *T*. *cordifolia* are affected by interspecific interactions with other plants. Till date no work has been undertaken to explore the the variations in phytochemicals of *T*. *cordifolia* due to inter-specific interactions with higher plants.

The available biomarker for quality control of *T*. *cordifolia* doesn’t seem to be sufficient and reliable due to variations in chemicals as result of its geographical location, climate and biotic interactions with higher plants. These issues have not been addressed during the slection of biomarkers. Therefore, to eplore the chemo-profiles of *T*. *cordifolia* and to identify reliable biomarkers, a highly sophisticated tool, high performance liquid chromatography coupled with quadrupole time of flight mass spectrometer (HPLC-ESI-QTOF-MS) has been used. This tool not only provides high mass accuracy and resolution of mass fragments but can yield empirical chemical formulae to facilitate the structural elucidation even without the use of reference standards^10^. Furthermore, statistical analysis of mass data can recognize important markers of plants grown in different conditions^11^. Thus, the current study was aimed to sepeficlly evaluate the alterations in the secondary metabolites of *T*. *cordifolia* co-occurred with other plants. In order to understand the comprehensive impact of biotic interactions on the chemo-profiles of *T*. *cordifolia*, the samples were collected and analyzed using HPLC-ESI-QTOF-MS. The data was analyzed using principal component analysis (PCA) and multivariate analysis followed by SNK post-hoc test with permutative (n = 50) Bonferroni FDR multiple testing correction to compare chemoprofiles among the groups. Hence, study was focused to explore the changes in the chemoprofiles due to interspecific interactions of *T*. *cordifolia* co-occurred with other higher plants.

## Results

### General characteristics of TCEs and HPLC method development

**Figure 1:**
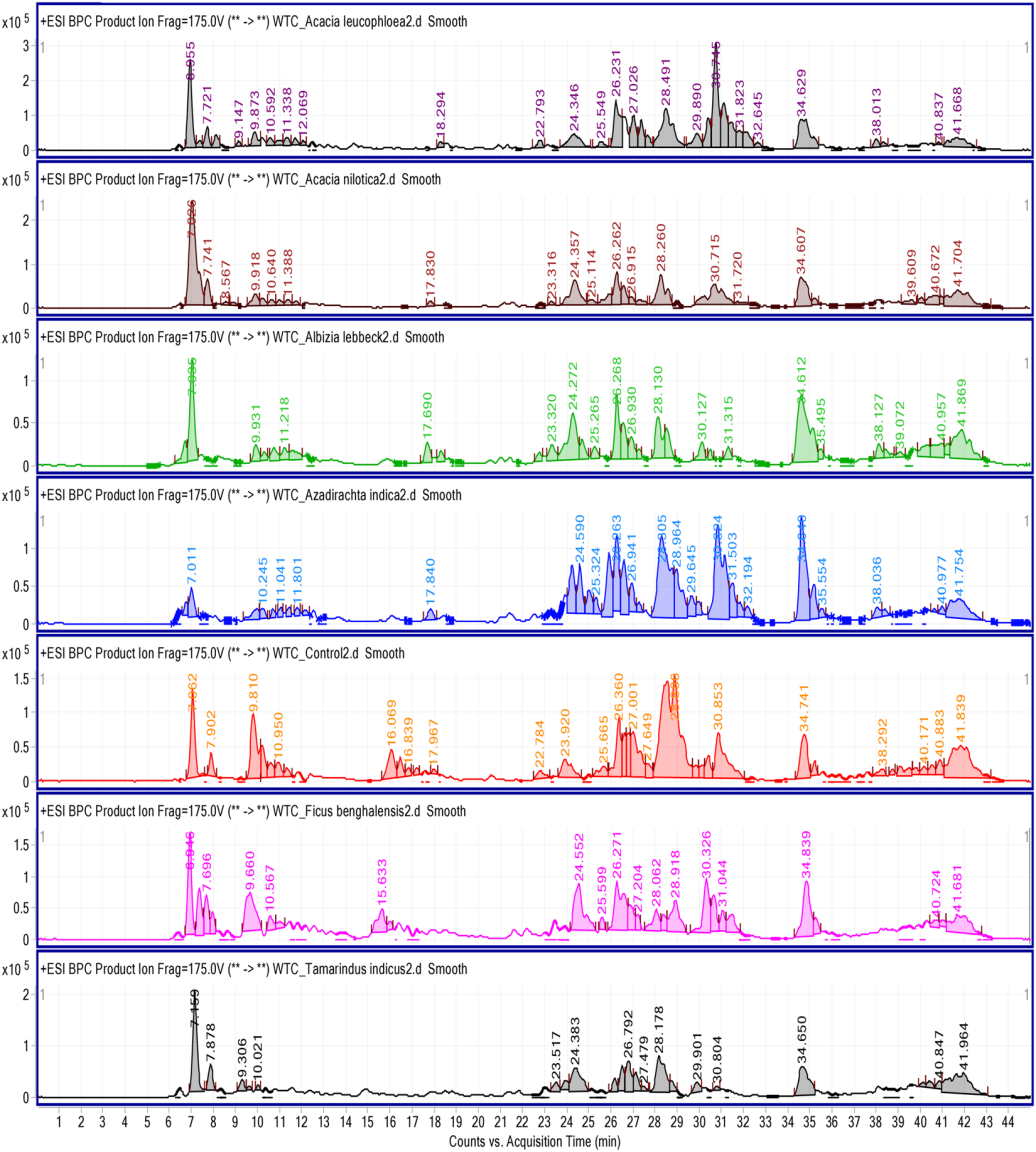
Base peak chromatograms of all seven groups extracted from total ion current chromatograms (TIC), showing visual differences among the chromatographic profiles of various groups. A, B, C, D, E, F and G represents the ALC, ANI, ALL, AIN, CON, FBG, TMI groups respectively.

Freshly prepared TCE was brown in color, pH 7.57, slightly bitter in taste and without any characteristic odour. Its specific gravity and viscosity were recorded to be 1.2 and 1.6cP. Our previous studies demonstrated that most of metabolites of *T*. *cordifolia* have been detected in positive ion polarity mode due to greater sensitivity to the signals as compared with the negative ion^5,12^. Therefore, total ion current chromatograms (TIC) of all the groups, i.e. control, AIN, ALL, ALC, ANI, TMI, and FBG were acquired in positive ion polarity mode. Water and acetonitrile with 0.1% formic acid selected as mobile phase as these solvents provided low background noise and better chromatographic peaks. Visual examination of base peaks of chromatograms extracted from TIC showed metabolite variations among the groups (Fig. 1). Intra and inter-day precision and accuracy were calculated by injecting a mixture of standards three times in a day for three consecutive days. Intra and inter-day precision was within 0.31 and 0.85%, while accuracies were more than 97.5 to 100%. Relative standard deviation (RSD) of repeatability of five different solutions was less than 1.57%. Recovery of the method was established by adding three different concentrations of reference standards to the crude extracts of *T*. *cordifolia*. The recovery of standards was found in between 98.07 to 100.07%, with RSD less than 2.63% (Table 1).

**Table 1:** Statistical results of inter and intraday precision, accuracy and recovery of five important compounds of *T*. *cordifolia*.

S. No.	Compounds	Intra-day (n = 9)		Inter-day (n = 9)		Repeatability (n = 9)	Recovery	
		Accuracy (RE%)	Precision (RSD%)	Accuracy (RE%)	Precision (RSD%)	RSD%	Mean Recovery (%)	RSD%
1.	Berberine	99.1	0.22	99.4	0.46	0.61	99.63	1.53
2.	Palmatine	97.4	0.17	100.4	0.43	1.57	99.31	1.46
3.	Jatrorrhizine	99.5	0.11	100.5	0.53	1.27	100.07	1.87
4.	Magnoflorine	98.7	0.16	99.7	0.85	0.95	99.21	2.63
5.	Choline	96.5	0.31	97.5	0.67	0.83	98.07	1.62

### Multivariate statistical analysis

**Figure 2:**
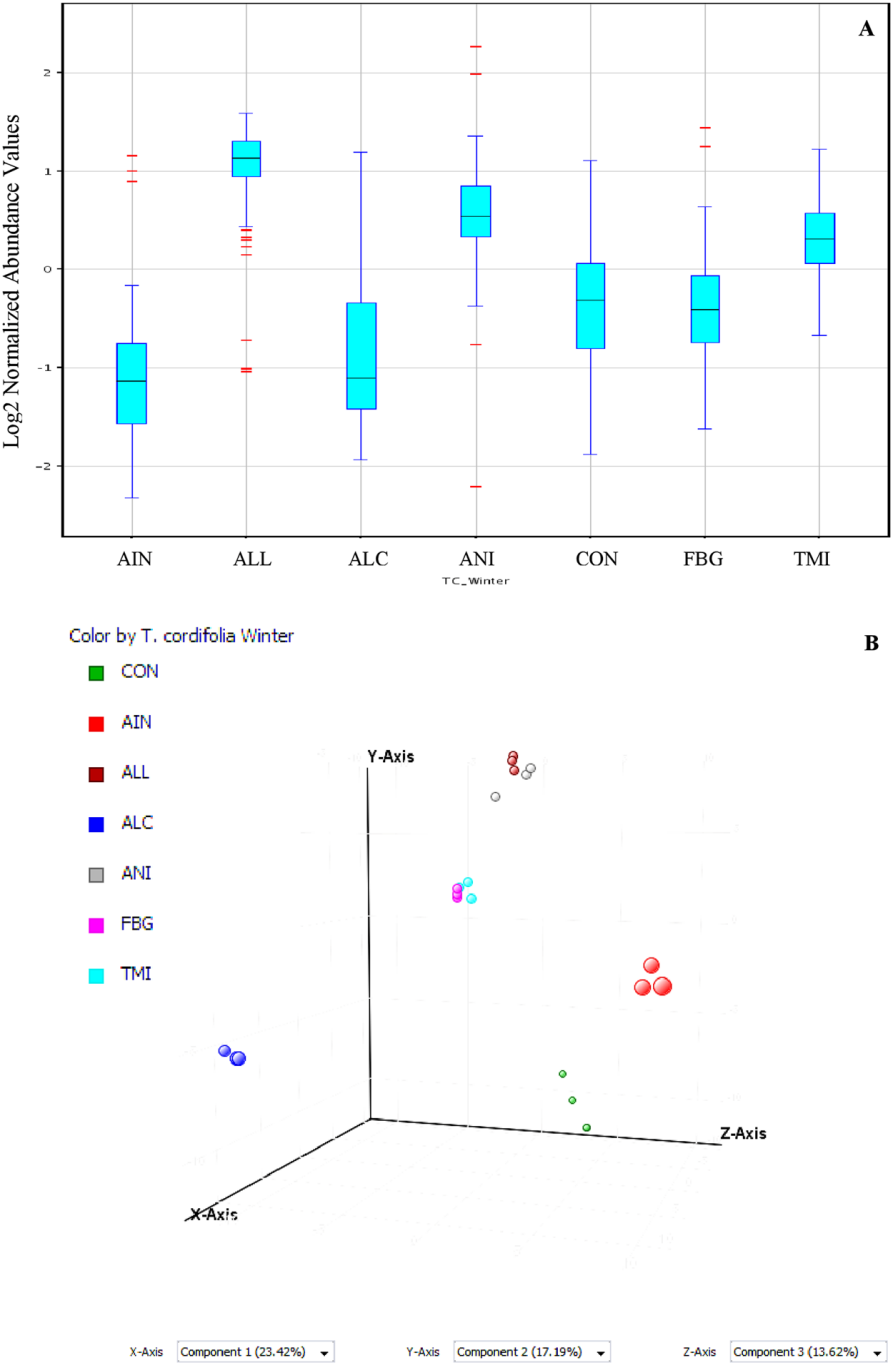
(**A**) Box Whisker plots of various groups after filtration, significance and fold change analysis showing high variations across the groups. (**B**) Principle component analysis score plot representing % variations among the metabolites of all seven groups where variability along the x, y and z axis are 23.42%, 17.19% and 13.62% respectively. AIN, ALC, ALL and ANI represents the distant groups showing highest variations.

Fifty nine major peaks were observed in the chromatogram when integrated. All the spectra were aligned using ion intensity, retention time (<0.2 min) and mass (<5 ppm) with the help of internal standards i.e. ions of *m/z* 296.15, 373.13, 311.13, 230.24, and 436.44 present universally in all the samples. Final data were normalized using Z-transforms. Data sets were subjected to one way ANOVA (p < 0.05), fold change (>2.0) and coefficient variation (>15%) analysis. ALL and ANI groups showed the highest number of down-regulated metabolites. Box Whisker plots of the data revealed least variability in the ALL group as compared to other samples. All the groups showed more variability in the upper quartile portion of Box Whisker plot (Fig. 2A). Supervised PCA was performed on all the datasets and visualized to check for outliers and classification trend among the samples (Table S1). Principal components have been extracted from the variables in the datasets. Statistical analysis involves principal component analysis projection to latent structures for identifying variation in spectral features of samples. PCA of 7 groups resulted in 1643 principal components. Each groups was observed to be distinct, forming their own cluster and lying far apart from each other. All the groups showed 23.42, 17.19 and 13.62% variations along the X, Y and Z axis respectively (Fig. 2B). Supervised PCA plot showed highest variations in AIN and ALC groups as compared to other groups. Further, data were subjected to multivariate analysis to identify and reveal differentially expressed metabolites in different groups. Initial analysis showed confidence in variability among the groups and the presence of distinct metabolites. Already established separation among the groups was sharpened by multivariate analysis. Data was further subjected to PLS-DA, SVM, NB, DT and NN classifiers for preparing respective classifier models^13^. Classifier models expressed confidence ranging from 1 to 0.896 (Table S2). All the models were trained for further prediction of unknown samples. Mass data files of different plant extracts and *T*. *cordifolia* (collected from different supporting trees) extracts were subjected to the trained models for classification and identification. A trained model has classified all the unknown samples and prediction measure were expressed as confidence measure. Trained model of PLS-DA, NB and DT failed to classify all known samples (Table S3), whereas SVM and NB classified all the extracts correctly and showed least r^2^ for the samples other than *T*. *cordifolia* (Table S2). Furthermore, SVM was found to be a better discrimination model and powerful classification tool in real-world applications due to expression of better confidence levels in *T*. *cordifolia* samples and least confidence in other extracts along with its excellent learning performance as reported earlier^14^. SVM data shown to have 1083 ranked metabolites from all the seven samples. Being best model, SVM data were subjected to further analysis to reduce the number of metabolites and to keep only significant variables.

### Differential metabolites of *T*. *cordifolia* due biotic interactions with different trees

**Figure 3:**
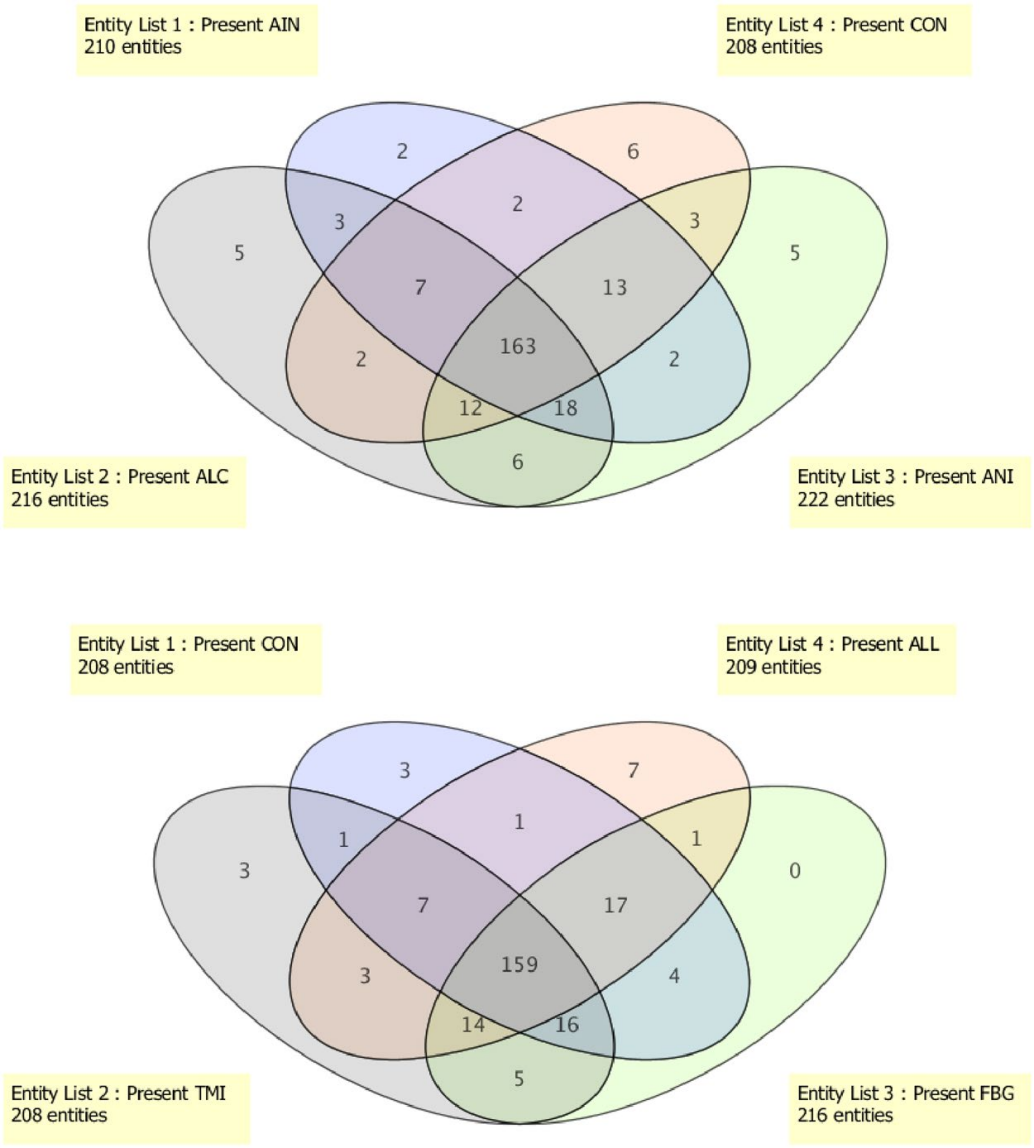
Venn diagram showing distribution of number of specific entities among seven different groups. The diagram shows overlapping and distinct metabolites indicated by the numbers in the intersections and circles, respectively.

**Figure 4:**
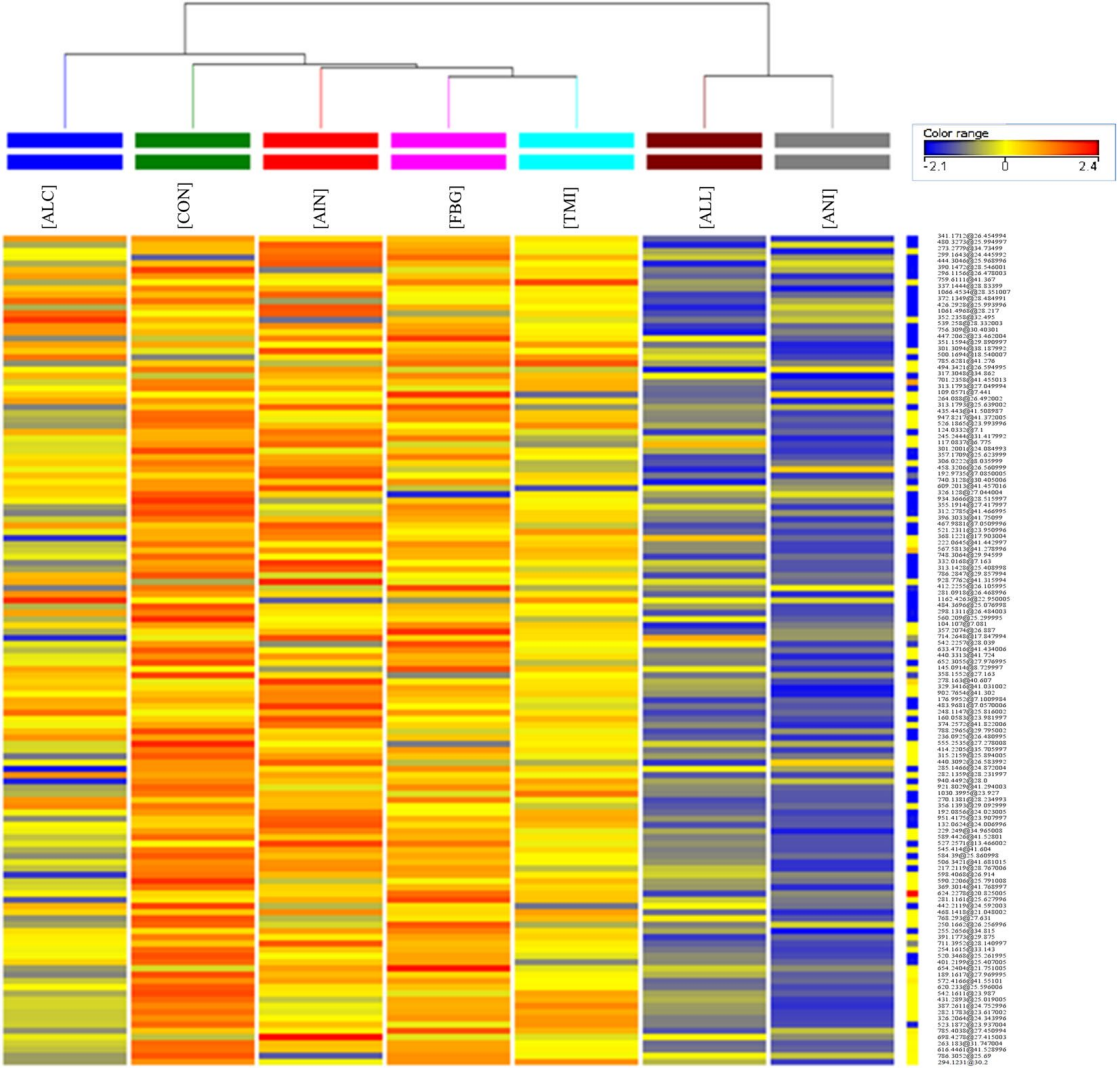
Spearman correlation heatmap of the metabolites without missing values, with compounds marked on the side of the map. Each row represents a sample and each column represents a metabolite feature. Color key indicates the normalized abundance of each metabolite expression value across the samples. The red and blue color indicates the highest and lowest metabolite expression values respectively. The figure clearly depicts the distinctness of ALL and ANI having lowest levels of most of the metabolites.

Venn diagrammatic representation of specific metabolites in respective groups showed the presence of 168, 167, 59, 126, 83, 80, and 150 differentially expressed metabolites in CON, AIN, ALL, ALC, ANI, TMI and FBG groups respectively. It showed the metabolites present in specific groups only, and/or may be due to missing values in data. Venn diagrammatic representation showed the presence of 6, 2, 7, 5, 5, 3, and 0 metabolites exclusively in CON, AIN, ALL, ALC, ANI, TMI and FBG groups, respectively (Fig. 3). It showed that 8-hydroxytinosporide and one unknown terpenoid were exclusively present in the AIN group while the control group contains 5-allyloxysalvigenin, trans-farnesol, reticuline, N-isovaleroylglycine and two unknown metabolites. The only ANI group contained palmatoside C, α-D-glucan, 5-aminovaleric acid and two unknown metabolites of higher molecular weight while, ALL group contained tinosporinone, baenzigeroside A, tinosinen, tinoridine and two unknown metabolites. The TMI group contains 11-hydroxymustakone along with two unknown metabolites (Table 2). Data were subjected to One-way ANOVA with SNK post-hoc test and asymptotic p values were computed with permutative (n = 50) Bonferroni FDR multiple testing correction. After analysis, 229 metabolites with p < 0.001 and fold change >2.0 were found to be differentially regulated. Missing values may originate from analytical, computational and biological backgrounds, therefore, cause problem in mass spectrometry data analysis. Mean imputation is the substitute of missing values. However, in the current study, entities with missing values were removed due to the high number of variables that reduced the number of discriminated metabolite to 133 (Table 3). The FBG group did not contain any specific metabolite. Observed mass differences and fragment ions are given in Table S3. Spearman correlation heat map of groups and metabolites without missing values is shown in Fig. 4. Spearman correlation heatmap analysis showed that TMI and FBG, control and ALC groups were close to each other, whereas ALL and ANI being more distant groups. Clusters of metabolites in red color and inter-spreading metabolites in blue color across the different groups are highly variable regions showing differential expression of metabolites in that particular group (Fig. 4). Differential metabolites without missing values were identified with the help of authentic standards and different standard databases [METLIN and MassBank] (https://metlin.scripps.edu/; http://www.massbank.jp/)^15,16^ by analyzing molecular formula, isotopic and fragmentation pattern (Table S4).

**Table 2:** Metabolites specifically and significantly present in various groups and represented in Venn diagram.

Groups	Compound/Mass	Mass	Retention Time
AIN	Unidentified terpenoid	457.36	41.69
	8-Hydroxytinosporide	390.1636	20.72
CON	952.7696	952.7696	41.34
	5-Allyloxysalvigenin	368.1217	20.66
	trans-Farnesol	222.0835	6.37
	636.6422	636.6422	41.28
	Reticuline (BP *m/z* 151)	329.1589	10.45
	N-Isovaleroylglycine	159.0412	8.05
ANI	775.2589	775.2589	41.47
	794.6717	794.6717	41.77
	Palmatoside C	636.6416	41.47
	(1,4)-α-D-glucan	180.0703	6.339
	5-Aminovaleric acid	117.0381	24.01
ALC	871.8031	871.8031	41.10
	389.1615	389.1615	20.99
	108.0251	108.0251	6.37
	604.2386	604.2386	24.83
	Tinocordifolioside	412.1304	30.13
ALL	5,6-Dihydrouracil	114.0181	8.06
	Tinoridine	315.216	25.16
	614.4143	614.4143	28.46
	Tinosporinone	329.1605	10.26
	Baenzigeroside A	520.3456	25.5
	Tinosinen	504.1888	6.38
	110.0407	110.0407	6.44
TMI	11-Hydroxymustakone	234.1723	27.30
	794.6717	794.6717	41.77
	880.6993	880.6993	41.41

**Table 3:** Fold change found in the metabolites in different groups in comparison to control [CON] group.

S. No.	Tentative Identification	RT	Mass	p (Corr)	AIN	ALC	ALL	ANI	FBG	TMI
1	Choline*	7.08	104.1070	6.79E−07	−1.8	−1.4	−6.2	−3.7	1.1	−1.3
2	Aminophenol*	7.44	109.0571	2.59E−06	−1.1	1.3	−2.5	1.1	3.1	−2.5
3	Betaine	6.77	117.0837	1.72E−05	1.3	−2.2	−1.2	−5.4	−2.2	−3.3
4	Phenylmethanethiol	7.10	124.0332	7.74E−15	1.4	1.0	−4.8	−3.8	−1.1	−1.8
5	Cinnamaldehyde	24.0	132.0624	2.25E−11	2.1	−1.2	−3.6	−3.4	−1.0	1.1
6	Isoquinoline N-oxide	8.72	145.0914	6.23E−09	−1.7	1.7	−1.1	−3.3	2.8	1.4
7	6-Methylcoumarin*	23.9	160.0583	3.46E−12	2.1	−1.2	−3.2	−3.8	−1.2	1.1
8	Sodium thiosalicylate*	7.10	176.9952	2.40E−12	1.2	−1.0	−5.2	−4.1	−1.2	−1.8
9	Acetamido-6-aminohexanoic acid	27.9	189.1617	7.70E−09	−1.8	−5.8	−4.4	−7.2	−2.2	−2.7
10	1,3 Dimethylpteridine-2,4-dione	24.0	192.0856	1.40E−14	1.2	−1.9	−4.4	−5.5	−1.8	−1.1
11	3-[4-(3-Aminopropylamino) butylamino]propanoicacid	28.7	217.2119	2.06E−13	−1.1	−3.0	−2.9	−6.9	−1.1	−2.9
12	Ethyl ferulate	41.4	222.0645	8.38E−07	−2.0	−3.2	−4.3	−6.8	−1.1	−1.6
13	Amino-tridecanoic acid*	34.9	229.2490	5.89E−09	1.2	−1.9	−2.8	−6.6	−1.0	−1.9
14	Glycosminine	26.4	236.0925	1.26E−12	−1.3	1.0	−4.3	−5.9	−1.2	−1.5
15	Haplopine*	31.4	245.2444	2.28E−09	1.2	−1.7	−2.0	−5.8	1.3	−2.5
16	Trimethyldecahydrophenanthren-2-ol	25.8	248.1147	1.30E−08	1.7	2.4	−2.8	−2.3	1.8	1.2
17	Tinocordifolin	26.2	250.1662	2.83E−09	−3.2	−4.2	−5.2	−3.3	1.0	−3.9
18	Chrysin*	33.1	254.1615	2.22E−08	1.7	−2.5	−5.4	−7.4	−1.5	−2.3
19	Palmitic amide	34.8	255.2656	1.41E−10	1.4	−1.0	−2.0	−5.1	1.7	−1.0
20	*Amyl p-butylaminobenzoate*	31.7	263.1830	1.88E−08	−1.7	−3.2	−5.7	−6.5	−1.4	−2.4
21	Neocryptotanshinone II	28.2	270.1381	1.93E−11	1.1	1.3	−3.8	−3.6	1.5	−1.4
22	13-Methyl-17−norabieta-15-ene-8-ylium	34.7	273.2779	3.18E−09	1.5	−1.4	−2.4	−5.6	1.2	−1.5
23	Alkaloid	26.4	281.0918	3.02E−15	−1.4	1.0	−4.3	−5.9	−1.2	−1.5
24	Carboxylic acid	25.6	281.1161	1.37E−06	1.4	−3.1	−1.5	−2.4	2.5	1.2
25	Coclaurine	24.8	285.1466	3.04E−15	−1.6	−7.8	−1.9	−3.1	1.0	−1.4
26	Magnoflorine-[(CH_3_)2NH]	26.4	296.1156	8.59E−16	−1.6	−1.0	−4.6	−6.3	−1.3	−1.5
27	N-Methylcoclaurine	24.4	299.1643	6.49E−14	6.3	2.4	1.8	1.4	6.0	3.8
28	Unknown ester (Floridimine type)	24.1	301.2001	1.65E−10	−3.3	−3.5	−4.5	−9.5	−2.1	−2.5
29	Sphinganine	38.1	301.3094	5.93E−05	3.2	1.6	−1.2	−2.9	1.8	1.1
30	(−)−Gallocatechin	8.03	306.0222	5.72E−05	−1.2	−1.6	−6.0	−4.6	−1.7	−3.3
31	3-Oxo-nonadecanoic acid	41.4	312.2785	1.68E−10	−3.8	−6.0	−5.3	−5.4	−1.6	−2.2
32	Feruloyltyramine*	25.4	313.1428	1.84E−14	1.3	−3.2	−3.3	−5.2	−1.1	−2.5
33	(+/−) Oblongine*	27.0	313.1793	2.99E−13	1.8	−2.8	−2.3	−2.1	1.9	−2.5
34	(+/−) Oblongine	25.6	313.1793	5.95E−14	−1.3	−2.1	−5.1	−6.7	−2.5	−1.3
35	Phytosphingosine	34.8	317.3048	3.61E−15	2.1	2.1	1.3	−3.2	2.7	1.9
36	Robinobiose	27.0	326.1280	3.00E−11	−2.0	−2.1	−4.4	−3.1	−9.4	−2.3
37	Dideoxysulphonated steroid	24.3	326.2064	7.72E−07	−1.8	−2.6	−4.1	−5.8	−1.0	−1.0
38	Icosasphinganine	41.0	329.3416	2.31E−05	1.6	1.2	−1.9	−4.7	1.4	1.0
39	Jatrorrhizine*	28.8	337.1444	3.71E−17	−1.3	−1.5	−3.8	−8.3	−1.7	−1.8
40	Magnoflorine [M+]	26.4	342.1712	2.78E−14	−1.5	1.0	−4.5	−6.1	−1.2	−1.5
41	8-Oxoberberine	29.8	351.1594	1.97E−15	−1.6	1.0	−3.6	−6.0	−1.2	1.0
42	Palmatine*	32.4	352.2358	1.64E−08	−3.6	2.1	−3.4	−2.1	−1.0	−1.8
43	Corydine methyl ether	27.4	355.1914	3.48E−11	−2.7	−4.4	−3.0	−7.9	−1.2	−2.3
44	N-Tetrahydropalmatine*	29.0	356.1393	2.21E−09	−1.5	−1.1	−5.9	−5.2	−2.1	−3.0
45	Isocorydine-N-oxide	25.6	357.1709	2.52E−11	1.0	−1.3	−4.6	−4.3	1.3	−1.5
46	Isoquinolone alkaloid	26.8	357.2074	3.19E−09	1.2	−1.9	−3.1	−2.2	2.8	1.0
47	Tinosporin	27.1	358.1552	6.08E−10	−2.5	−2.1	−6.8	−5.4	−6.0	−3.0
48	Glucoside	17.9	368.1221	3.72E−08	1.2	−5.4	−1.1	−3.8	−1.2	−1.3
49	Pentamethoxyflavone	28.4	372.1349	2.25E−14	−3.9	1.0	−6.3	−3.8	−2.4	−1.9
50	Palmarin	41.8	374.2572	7.34E−06	1.4	−1.8	−3.2	−5.7	−1.3	−1.3
51	Steroidal Compound	24.7	387.2611	1.55E−07	−2.2	−2.5	−3.5	−6.7	−1.2	1.0
52	Salvinorin B	28.5	390.1472	7.17E−11	−6.7	−2.1	−6.6	−4.8	−3.6	−2.5
53	Stigmastan-3,5-diene	41.7	396.3033	1.98E−07	−1.5	−2.8	−4.4	−7.2	−1.3	−1.7
54	Tinocordifolioside	26.1	412.2255	3.53E−10	−3.0	−4.8	−4.0	−2.4	1.3	−3.4
55	Lycopene derivative	35.7	414.2205	5.05E−09	−1.6	−2.7	−4.8	−6.9	−1.2	−1.8
56	Cycloeucalenol	25.9	426.2928	5.57E−16	1.8	−2.3	−5.3	−1.8	1.0	−1.4
57	Glucoside	24.5	442.2119	5.21E−14	−4.5	−1.9	−3.1	−6.8	−2.5	−6.7
58	3β,5α,14α−Trihydroxyergosta-7,22-dien-6-one	25.9	444.3046	1.02E−16	1.8	−2.3	−5.4	−1.7	1.0	−1.4
59	Glucoside of m/z 286	23.4	447.2062	6.22E−15	2.8	−1.4	1.4	−1.9	4.2	1.5
60	Cycloartane-24,25-diol-3-one	26.5	458.3206	1.17E−11	1.4	−1.9	−6.6	−1.3	−2.4	−2.6
61	20-Hydroxyecdysone*	25.9	480.3273	6.75E−14	1.7	−2.3	−5.4	−1.8	1.0	−1.4
62	Auricularine	26.5	494.3421	4.19E−08	−1.2	−2.1	−8.6	−1.8	−2.7	−2.9
63	Tinosporaside	18.5	500.1694	4.34E−12	2.5	4.5	1.7	−1.4	3.8	3.7
64	Baenzigeroside A	25.2	520.3468	4.63E−11	−2.9	−2.9	−5.4	−8.1	−1.6	−3.3
65	O-Glucoside derivative	28.0	542.2257	4.87E−10	−3.5	−2.1	−1.6	−5.5	1.5	−1.8
66	O-Glucoside derivative	25.2	560.2090	2.31E−11	−3.5	−5.3	−3.8	−8.8	−2.9	−5.1
67	Isotanshinone II *	30.2	294.1231	0.048351	3.3	5.4	3.4	5.2	2.9	3.2
68	Cordifolide A	26.9	598.4068	2.64E−09	−1.5	−7.3	−2.9	−4.5	−1.3	−2.0
69	Isoquinoline alkaloid	41.5	616.4461	2.59E−04	−1.7	−3.4	−5.2	−5.8	−1.3	−2.2
70	Saponin glycoside	25.6	620.2330	5.25E−09	−2.3	−2.6	−6.1	−7.8	−1.9	−2.6
71	Chrysoeriol C-hexoside O-hexoside	20.8	624.2278	0.034911	−1.0	−1.1	−3.8	−2.4	1.8	1.0
72	Unknown cinnamic derivative	41.4	633.4716	2.54E−07	−2.6	−3.9	−5.8	−6.0	−1.3	−2.8
73	Glucoside of m/z 493	21.7	654.2404	4.05E−07	2.2	−1.4	−1.0	−1.5	4.9	1.0
74	5-Allyloxysalvigenin derivative	17.8	714.2648	1.18E−09	1.9	−1.3	−3.5	−4.1	1.0	−1.2
75	Unknown of *m/z* 184.08	41.2	785.6281	0.001743	−1.3	−2.6	−3.8	−2.0	1.9	1.0
76	Diosgenin 3-[glucosyl-(1->4)-rhamnosyl-(1->4)-[rhamnosyl-(1->2)]-glucoside	23.9	1030.3995	3.42E−13	−1.4	−3.3	−4.4	−5.8	−2.6	1.0

Compounds have been identified using mass error >5 ppm and fragment ions and arranged according to their retention time.

Note: *Compound identified with standard compound; Italics: Compounds identified by structure elucidation with help of MSC (Agilent Technologies) and Metfrag software; all other compounds were identified by comparing MS/MS spectra with standard libraries from METLIN and MassBank. Unidentified compounds are represented as unknown or by formula.

### Differential metabolites in AIN group

Along with 8-hydroxytinosporide and one unknown terpenoid present exclusively in this group. Protonated molecule [M + H]^+^ at *m/z* 391.164 was identified as 8-hydroxytinosporide due the presence of characteristic mass ions at *m/z* 373 [M − H_2_O]^+^, 355 [M − H_2_O]^+^, 347 [M-CO_2_]^+^, 253 and 123 as reported earlier^17^. Statistical analysis showed presence of 18 compounds up-regulated by >2.0 fold in AIN group. High levels (>3 fold) of borapetosides D, N-methylcoclaurine, isotanshinone IIA, peonidin and 5-allyloxysalvigenin derivative were found in this group (Tables 3 and S2). Borapetoside D is a clerodane-type furanoditerpene containing two D-glucopyranose units, hence consecutive loss of 162 Da from parent ion [M + H]^+^ of *m/z* 698 generate positive ion fragments at *m/z* 375 that further generate ions at *m/z* 357 [M -Glu –Glu -H_2_O]^+^, 343 (CH_4_), 265, 206, 185, 159, 149, 121 and 105 as reported earlier^18^. N-Methylcoclaurine resolved at 24.4 ± 0.2 min in all the groups and parent ion [M + H]^+^ at *m/z* 299.16 further produced positive ions at *m/z* 282, 271, 257 due to loss of H_2_O, CH_3_OH and CH_3_. It also formed positive ions at *m/z* 192, 163, 121, 119 and 107 in which ion of *m/z* 163 is characteristic ion of N-methylcoclaurine formed from ion of *m/z* 178 due to loss of methyl group. Protonated parent ion [M + H]^+^ at *m/z* 295 resolved at 30.2 ± 0.2 min was identified as isotanshinone II as it afforded mass fragments at *m/z* 277 [M-H_2_O]^+^, 267 [M-CO]^+^, 249 [M-H_2_O-CO]^+^, 239 [-C_3_H_4_O]^+^, 187, 159, and 111. Parent ion [M + H]^+^ of *m/z* 301 showed predominant product ions at *m/z* 283 [M-H_2_O]^+^, and other ions at *m/z* 269, 257, 229, 137, 129 that match with standard spectra of peonidin. Protonated molecule [M + H]^+^ at *m/z* 714.264 showed product ions at *m/z* 369, 351, 337, 329 and 311, the characteristic ions of 5-allyloxysalvigenin, hence compound was identified as derivative of 5-allyloxysalvigenin.

Other up-regulated compounds whose level was found to be >2 fold but <3 fold were tinosporaside, jatrorrhizine, glucoside 493, 1-{hydroxy[2-(trimethylammonio) ethyl]amino}-1-oxo-2-dodecanaminium, 6-methylcoumarin, and cinnamaldehyde (Table 3). A protonated molecule [M + H]^+^ at *m/z* 500.169 resolved at 18.5 min was identified as tinosporaside due the presence of dominant positive product ions at *m/z* 339 [M-162] due loss of glucose moiety, 323 due loss of H_2_O and other ion at *m/z* 317, 207, 137 and 121. The [M + H]^+^ ion of Jatrorrhizine was observed at *m/z* 338.1387 and RT 28.8 ± 0.2. It produced fragment ions at *m/z* 323, *m/z* 308, *m/z* 295 and *m/z* 294 due to sequential loss of [M-CH_3_]^+^, [M-CH_3_-CH_3_]^+^, [M-CH_3_-CO]^+^ and [M-CH_4_-CO]^+^ ions. The presence of [M-CH_3_-H]^+^ ions at *m/z* 322 confirm the presence of methoxyl groups at C9–C10 positions (Fig. S4). The [M + H]^+^ ion at *m/z* 655 was resolved at 21.7 min and identified as unknown glucoside, as it afforded dominant ion at *m/z* 493 due to neutral loss of 162 Da of glucose moiety. Precursor ion *m/z* 161 generate products at *m/z* 133 and 105 due to consecutive loss of CO, hence presumed as 6-methylcoumarin. Cinnamaldehyde was identified as precursor ion [M + H]^+^ at *m/z* 133 forms product ions at *m/z* 115 and 105 due to loss of H_2_O and CO.

Up-regulated metabolites having fold change <2.0 as compared to control but >2.0 as compared to other groups were β,5α,14α-trihydroxyergosta-7,22-dien-6-one, cycloeucalenol, oblongine, 20-hydroxyecdysone, phenylmethanethiol, and cycloartane-24,25-diol-3-one (Table 3). The precursor ion scan and/or the low-energy CID-MS/MS of the protonated molecule at *m/z* 445 (precursor ion) afforded the following products ion at *m/z* 426 [M-H_2_O]^+^, 411 [M-H_2_O -CH_3_]^+^, 408 [M -2H_2_O]^+^, 393 [M -2H_2_O -CH_3_]^+ •^, 390 [M -3H_2_O]^+^ that lead to its identification as β,5α,14α- trihydroxyergosta-7,22-dien-6-one. The protonated molecule [M + H]^+^ at *m/z* 427 afforded product ions at *m/z* 409, 393, 383, 367, 353, 343, 325, 301, 233 and 125, when matched with standard library it was identified as cycloeucalenol with a confidence score of 97.6. Precursor ion [M + H]^+^ at *m/z* 315 breakdown into positive product ion at 297 [M-H_2_O]^+^, 287 [M-CO]^+^], 271 [M-26 Da aromatic character], 257, 206, 178, 119 and 107. These product ions matched with standard spectra of oblongine with confidence score 93. The precursor ion [M + H]^+^ at *m/z* 480 resolved at 25.9 min was identified as 20-hydroxyecdysone due to presence of product ions at *m/z* 462 and 444 formed due to loss of H_2_O molecules, and other characteristic ions at *m/z* 429, 426, 411, 408, 393, 363, 345, 344, 328, 327, 300, 145, and 143. The parent ion [M + H]^+^ at *m/z* 125 showed fragment ion at *m/z* 109 and identified as phenylmethanethiol. Precursor ion [M + H]^+^ at *m/z* 459 resolved at 26.5 min afforded product ions at *m/z* 441, 423 (due consecutive loss of H_2_O molecules), 313 and 175 as described earlier and identified as cycloartane-24,25-diol-3-one^19^.

Palmarin, feruloyltyramine, betaine, 1,3 dimethylpteridine-2,4-dione, sodium thiosalicylate, haplopine, isoquinolone alkaloid, amino-tridecanoic acid, glucoside of 207, tetramethylazobenzene, scalar-17(25)-en-19-ol were identified metabolite having fold change <1.5 as compared to control but have significant differential levels when compared to other groups (Table 3). The precursor ion scan of the protonated molecule at *m/z* 375 (precursor ion) afforded the following products ion at *m/z* 357 [M-H_2_O]^+^, [M-CO]^+^, 329 [M-CO, 2H_2_O]^+^, 307, 279, 165, and 125 that lead to its identification as palmarin. It was verified from Metlin standard database. Protonated molecule [M + H]^+^ resolved at 25.4 min of *m/z* 314 was identified as feruloyltyramine when compared with synthetic standard library as it yielded the fragment ions at *m/z* 296 [M-H_2_O]^+^, 284, 270. It yielded characteristic ion of primary acylium at *m/z* 177 which, further yielded fragment ions at *m/z* 149, 145, and 117. Betaine (*m/z* 118) was identified as produced identical spectra with synthetic betaine and resolution at 6.77 min. The precursor ion scan and/or the low-energy CID-MS/MS of the protonated precursor ion at *m/z* 193 resolved at 24 min afforded the products ion at *m/z* 177 [M-CH_3_]^+ •^, 161[M-2CH_3_]^+ •^, 133[M-2CH_3_-CO]^+ •^, and [M-2CH_3_ –CO -H_2_O]^+•^ was identified as 1,3 dimethylpteridine-2,4-dione using standard database. Parent ion [M + H]^+^ at *m/z* 177.99 was identified as sodium thiosalicylate as it yielded product ion at *m/z* 137, 133 and 105 using the standard database. Protonated molecule [M + H]^+^ at *m/z* 246 resolved at 31.4 min and yielded product ions at *m/z* 228 [M-H_2_O]^+^, 218 [M-CO]^+^, 214, 202, 186, 174 and 142 matched with haplopine in standard database Metlin with a confidence score of 97.23. Precursor ion [M + H]^+^ at *m/z* 358 resolved at 26.8 min afforded characteristic fragments ions at *m/z* 192, 151 and 137 of isoquinolone, however it could not be identified hence, named as isoquinolone alkaloid. Precursor ion [M + H]^+^ at *m/z* 230 yielded product ions of 212 and 195 due neutral loss of H_2_O followed by NH_4_. It also yielded characteristic ions of *m/z* 184, 167, 155 related to tridecanoic acid; hence it was identified as amino-tridecanoic acid. A precursor ion [M + H]^+^ at *m/z* 369 afforded product at *m/z* 207 due to loss of glucose moiety, further identification could not be established, hence named as glucoside of 207. Parent ion [M + H]^+^ at *m/z* 358 resolved at 25.6 min yielded product ions at *m/z* 343 [M-CH_3_]^+^, 327[M-2CH_3_]^+ •^, 272, 259, 191, 163, 137, and 123, hence, identified as scalar-17(25)-en-19-ol as reported previously^20^.

### Differential metabolites in ALC group

In this group, compounds of 108, 389, 604, 871 Da and tinocordifolioside were identified exclusively. Precursor ion scan of the protonated molecule at *m/z* 413 afforded the products ion at *m/z* 251 die loss of 162 Da (glucose moiety), hence identified as tinocordifolioside. Other compounds uniquely present in this group could not be identified. ALC group also showed elevated levels (>2.0 fold) of isotanshinone IIA, tinosporaside, saponin, N-methylcoclaurine (described in AIN group), dehydrocorybulbine, and palmatine (Table 3). The compound resolved at 32.4 min of *m/z* 353 [M + H]^+^ yielded the fragment ions at *m/z* 337 [M -CH_4_]^+^, 321 [M -2CH_4_]^+^, 308, 294 and 279 was unambiguously assigned as dehydrocorybulbine after comparing with standard spectra. The [M + H]^+^ ion of palmatine was observed at *m/z* 352. Low-energy CID-MS/MS afforded the prominent product at *m/z* 337 [M-CH_3_]^+ •^ due to the loss of methyl radical. It produced ions product ions at *m/z* 322 and 308 due to sequential losses of hydrogen radical and CO. Product ions including others at *m/z* 294, 278, 264 and 250 were equivalents to mass spectra obtained from synthetic palmatine (Fig. S4), hence compound was identified as palmatine.

Compound found to present in higher amounts as compared to other groups but < 2.0 fold as compared to CON group were (9Z,14Z)-octadeca-9,14-dien-6-ynoic acid, isoquinoline N-oxide, peonidin, N-[3-(cyclohexylamino)-4-methoxyphenyl] acetamide, tetramethylazobenzene, aminophenol, borapetosides D and tetrahydro-4-acetyl-1-methyl-7-(4-pyridinyl)-3(2 H) isoquinolinone (Table 3). Precursor ion resolved at 8.72 and at *m/z* 146 [M + H]^+^ yielded major product ion at *m/z* 129 and 128 due to loss of N linked oxygen as H_2_O and 102 due Diels-Alder fragmentation, identified as isoquinoline N-oxide. Aminophenol was identified using isomeric pattern of precursor ion keeping mass error > 5 ppm. Precursor ion [M + H]^+^ at *m/z* 699 resolved at 27.4 min yielded product ion at *m/z* 537 due loss of glucose moiety (162 Da) that further yielded peak at *m/z* 121 due to the furan ring conjunction indicated that these compounds are furanoid diterpenes with an oxygen at C-12 and an angular methyl group at C-9. It also afforded product ions at *m/z* 375, 357, 343, 265, 231, 205, 185, 159, 149, 121 and 133 which led its identification as borapetoside D.

### Differential metabolites in FBG group

Venn diagram analysis didn’t show presence of any specific compound in FBG group. However, *T*. *cordifolia* supported by *F*. *bengalensis* found to have > 2.0 fold increased level of N-methylcoclaurine, glucosides of 493 and 286 Da compounds, tinosporaside, aminophenol, isotanshinone II, isoquinoline N-oxide, isoquinolone alkaloid and one unknown carboxylic acid derivative (Table 3). Other identified compounds having levels < 2.0 fold as compared to CON group but significant altered levels as compared to other groups includes oblongine, peonidin, chrysoeriol C-hexoside O-hexoside, palmitic amide, tetramethylazobenzene, 5-allyloxysalvigenin derivative, haplopine, tinocordifolioside, borapetoside D and choline (Table 3). The protonated molecule [M + H]^+^ observed at *m/z* 625 corresponding to chrysoeriol C-hexoside O-hexoside as its fragment ions at 463 and 445 were assigned as loss of C-glucose and O-glucose moieties. Further, product ions at *m/z* 313, 295 and 163 confirmed its identity as chrysoeriol. Precursor ion [M + H]^+^ at *m/z* 714 resolved at 17.8 min afforded product ions at *m/z* 535, 369, 351, 337, 329 and 311. Last four peaks were found to match with standard mass spectra of allyloxysalvigenin; hence it was named as allyloxysalvigenin derivative. Precursor ion resolved at 25.6 min and [M + H]^+^ at *m/z* 358 yielded product ions at *m/z* 343 [M-CH_3_]^+ •^, 313 [M-CH_3_ -NH_2_]^+^, 295 [-H_2_O]^+^, 281, 263 and 133, indicated presence of N-methyl and however it could not be identified. Precursor ion [M + H]^+^ at *m/z* 246 resolve at 31.4 yielded product ions at *m/z* 231 [M -CH_3_]^+ •^, 230, 228 [M-H_2_O]^+^, 216 [M -2CH_3_]^+ •^, 218, 214, 202, 186, and 174 was established as haplopine when compared with standard.

### Differential metabolites in TMI group

11-Hydroxymustakone (*m/z* = 235.1723) and two unknown compounds of *m/z* 795 and 881 were exclusively present in this group only. Precursor ion [M + H]^+^ at *m/z* 235 afforded product ions at *m/z* 217 [M -H_2_O]^+^, 189 [M -H_2_O -CO]^+^, 175 [M -C_3_H_6_]^+^ 161, 131 and 107 corresponding unambiguously to 11-hydroxymustakone when compared with standard spectra. *T*. *cordifolia* co-occurred with *T*. *indica* didn’t show significant increased levels of metabolites (Table 2). However, isotanshinone IIA, tinosporaside and N-methylcoclaurine were found to be increased as compared to control. Down-regulated metabolites includes cordifolide A, 3-oxo-nonadecanoic acid, glaucine, cyclohexane-1,3-dione, robinobiose, isonicotonic acid, amyl p-butylaminobenzoate, aminophenol, oblongine, haplopine, feruloyltyramine, (S)-6-O-methylnorlaudanosoline, sulphonated steroid, cycloartane-3-oxo-24,25-diol, phosphatidylcholine, myricetin, makisterone A, N-tetrahydropalmatine, tinosporin, palmatoside C, gallocatechin, betaine, baenzigeroside B, tinocordifolioside and tinocordifolin (Table 3).

### Differential metabolites in ALL group

ALL group exclusively contains 5, 6-dihydrouracil, tinoridine, baenzigeroside A, tinosinen and unknown compounds at *m/z* 615 and 111. After initial analysis, 5,6 dihydrouracil was identified using mass range from *m/z* 50 to 100 in Q-TOF-MS as it yielded characteristic product ions at *m/z* 73, 55, and 56 due to loss of H_2_CCO, NH_2_, CONH_2_ and formation of NH_2_CONH_3_^+^. Precursor ion [M + H]^+^ at *m/z* 316 yielded product ions at 271 [M -C_2_H_6_O]^+^, 198 [M -C_8_H_9_N]^+^, 152 [198 -C_2_H_6_O]^+^, and 120 [M -C_9_H_11_NO_2_S]^+^, hence structure established to tinoridine. Parent ion [M + H]^+^ at *m/z* 330 yielded product ions at *m/z* 314 [M -CH_3_]^+^, 298 [M -2CH_3_]+, 168 and 151, however structure could not be established. Precursor ion [M + H]^+^ at *m/z* 521 yielded product ions at *m/z* 359 [M -Glu], 313, 247, 213, 203, 165, 151, 133, 119, and 105 similar to baenzigeroside A, hence compound was established as baenzigeroside A. Precursor ion [M + H]^+^ at *m/z* 505 yielded product ions at *m/z* 373 ad 343 due to loss of pentose and hexose moieties. Further ion at *m/z* 343 yielded 325 [M -H_2_O]^+^, 311 [M –CH_3_]^+^, 293 [–H_2_O], 275 [–H_2_O], 211, 197, 163 and 133 unambiguously established the structure of tinosinen.

*T*. *cordifolia* grown on *A*. *lebbeck* (ALL) didn’t show elevated levels of metabolites after elimination of the metabolites without missing values (Table 3). Isotanshinone IIA, N-methylcoclaurine, tinosporaside, borapetosides D were found to be up-regulated as compared with control. However, in comparison of other groups these compounds were under expressed in this group. In Table 3, most of the compounds were found to be significantly suppressed in *T*. *cordifolia* climbed on ALL, which is the highest number of suppressed metabolites among all the groups. Some of known metabolites i.e. haplopine, oblongine, cordifolide A, glaucine, 6-methylcoumarin, palmarin, feruloyltyramine, dehydrocorybulbine, palmatine, cinnamaldehyde, jatrorrhizine, magnoflorine, tanshindiol, tinocordifolin, cycloeucalenol, dideoxyecdysone, 20-hydroxyecdysone, palmatoside C, N-tetrahydropalmatine, tinosporin, and makisterone A were among the down-regulated metabolites in this group.

### Differential metabolites in ANI group

In this group α-D-glucan, 5-aminovaleric acid, Palmatoside C and two unidentified compounds at *m/z* 775 and 795 were found to be uniquely present. Precursor ion [M + H]^+^ at *m/z* 181 showed product ions as of in glucose when compared standard, hence named α-D-glucan (breakdown unit of glucans). Protonated molecule [M + H]+ at *m/z* 118 produce product ions at *m/z* 101 and 100 due to loss of NH_3_ and H_2_O molecules, therefore identified as 5-aminovaleric acid also confirmed using the monoisotopic mass error <5 ppm. Biotic interactions of *A*. *nilotica* (ANI) with *T*. *cordifolia* displayed elevated levels of isotanshinone IIA and (9Z,14Z)-octadeca-9,14-dien-6-ynoic acid (Table 3). Most of the other metabolites were found to be down-regulated as in case of ALL group that includes oblongine, palmatine, tinocordifolin, cinnamaldehyde, feruloyltyramine, magnoflorine, and jatrorrhizine well-known metabolites from *T*. *cordifolia*.

### Upregulated metabolites in CON group

Control group contain specific metabolites namely 5-allyloxysalvigenin, trans-farnesol, reticuline (BP *m/z* 151) and two unidentified metabolites of *m/z* 953 and 637. Precursor ion [M + H]^+^ at *m/z* 369 resolved at 20.7 min yielded product ions at 353 [M –CH_4_]^+^, 351 [[M –H_2_O]^+^, 321 [M -CH_3_OH]^+^, 323 [M –H_2_O –CO]^+^, 311, 297, 285, 269 and 107, a typical fingerprint of allyloxysalvigenin. Parent ion [M + H]^+^ at *m/z* 223 resolved at 6.4 min yielded product ion of *m/z* 205 due to loss of water molecule, 163 due to loss of CH_3_CH=CH_2_, 149 due to loss of CH_4_, 135 due to loss of CH_3_, 123, 121 due to loss of CH_3_, 109 and 107 again due to loss of CH_3_ established its structure as trans-farnesol. Protonated molecule [M + H]^+^ resolved at 10.5 min and at *m/z* 330 afforded product ion at *m/z* 314, 301, 298, 287, 244, 192, 151, 149, 137 and 123. These are the characteristic ion of reticuline. It was further confirmed by the presence of the most abundant ion of *m/z* 192 which formed due to loss of 138 Da through four member ring arrangements and the ion of *m/z* 137 formed due to benzylic cleavage.

A number of other metabolites were also found to be up-regulated in CON group as compared to other groups (Table 3) and some important ones are described here. 8-Oxoberberine [M + H]^+^ ion at *m/z* 352.1594 was identified due to sequentially loss of CH_3_, CO and CH_4_ generated ions at *m/z* 337, 308 and 292. The precursor ion [M + H]^+^ at *m/z* 356 yielded product ions at *m/z* 341[M -CH_3_]^+^, 326 [M -2CH_3_]^+^, 313 [M -CH_3_ -CO]^+^, 311, 279, 265, 206, 192 [prominent peak due ring cleavage] and 133 has been identified as tetrahydropalmatine using reference standard. Another parent ion at *m/z* 342 [M]^+^ generated strong ions peak at *m/z* 297 due to loss of dimethylamine molecule [(CH_3_)_2_NH, at *m/z* 282, 265 and 237 due to loss of methyl radical, methanol and CO, respectively. These are the characteristic feature of protoberberine alkaloids; hence compound was unambiguously identified as magnoflorine (Fig. S4). Two precursor ions [M + H]^+^ at *m/z* 297 and 265 were identified as degradation products of magnoflorine, as these afforded the peaks of magnoflorine -[(CH_3_)_2_NH]+ and magnoflorine -[(CH_3_)_2_NH]-CH_3_OH-CH_3_]^+^. The precursor ions [M + H]^+^ at *m/z* 297 generated product ions at *m/z* 265, 237, 219, 207 and 179 and [M + H]^+^ *m/z* 265 generated the product ions at *m/z* 237, 219, 207 and 179 as in case of magnoflorine. Precursor ion [M + H]^+^ at *m/z* 355 generated product ions at *m/z* 311 [M -CO_2_]^+^, 296 [M –CO -CH_3_]^+^, 279 [*m/z* 311 - CH_3_OH]^+^, 265 [-CH_3_]^+^, 251 [-CH_3_]^+^, 229, 215, 192, and 104, just like magnoflorine with addition methyl group, hence compound was identified as corydine methyl ether. Precursor ion [M + H]^+^ at *m/z* 314 resolved at 25.6 min yielded product ions at *m/z* 299 [M-CH_3_]^+^, 281 [M -CH_3_ –H_2_O] + , 269 [M -(CH_3_)2NH]^+^, 237 [*m/z* 269 –CH_3_OH]^+^, 209, 192 [M– Isoquinoline], 175 [*m/z* 269 –C_6_H_6_], 143, 121, 107 [Benzylic cleavage], hence, it was identified as oblongine. Protonated molecule [M + H]^+^ at *m/z* 358 resolved at 26.8 min generated products ion at *m/z* 313 [M-CH_2_CH_2_OH]^+^, 299, 192, 151, 137. These are the characteristic ions of isoquinolone alkaloids as the product ion at *m/z* 299 formed by loss of CH_3_NH_2_, *m/z* 192 and 137 represent the isoquinoline and the benzylic cleavage fragment as discussed in case of oblongine, hence compound was named isoquinoline alkaloid. Precursor ion [M + H]^+^ at *m/z* 295 generated ion *m/z* 277, 253, 249 and 221 due to loss of H_2_O, CH=CHCH_3_, H_2_O and CO, was identified as isotanshinone IIA by comparing with standard spectra. Structure of precursor ion [M + H]^+^ at *m/z* 702 resolved at 41.4 min could not be established but it afforded product ions at *m/z* 563, 445, 429, 371, 355, 341, 297, 281, 267, 223, 207 and 149, later one are similar ions to tanshinone, hence named as tanshinol derivative. Protonated molecule [M + H]^+^ resolved at 24.1 min and at *m/z* 302 yielded the product ions at *m/z* 284 [M –H_2_O], 256 [M -C_2_H_5_OH], 161, 123 and 102, indicating it as an ester derivative, however exact structure could not be established.

Precursor ion [M + H]^+^ at *m/z* 359 yielded product ions at *m/z* 341 [M –H_2_O]^+^, 327, 309 [–H_2_O]^+^, 295 [–CH_2_]^+^, 281 [*m/z* 309 -CO]^+^, 263 [–H_2_O]^+^, 247, 235 [*m/z* 263 –CO], 215, 189, 171 [*m/z* 215–CO_2_], 143 [-CO], 137, 105 showed the presence of six oxygens and furan ring, hence assigned as tinosporin B comparing with reference spectra. Precursor ion [M + H]^+^ at *m/z* 413 yielded product ion at *m/z* 251 due to loss of glucose moiety of 162 Da. Further it generated molecular ions at *m/z* 233 [-H_2_O]^+^, 215 [-H_2_O]^+^, 175 [Aromatic], 159, 147 [-CO], 135, 121 and 109, the characteristic ions of tinocordifolioside. The molecular formula of molecular ion peak at *m/z* 599.4156 was calculated as C_28_H_38_O_12_S that further generated product ions at *m/z* 581, 535, 469, 385, 361, 298, 247, 193, 167 and 102. The product ion at *m/z* 361 was formed due to loss of C_8_H_14_O_6_S. Comparing the fragmentation, it was tentatively assigned as cordifolide A. The precursor ion [M + H]^+^ at *m/z* 251 generated distinctive product ion at *m/z* 233 [M -H_2_O]^+^, [M -2H_2_O -CO]^+^ 175 [*m/z* 203 –CO]^+^, 159 [-CH_4_]^+^, 145, 133 [-HCCH]^+^, 117[-CH_4_]^+^, which were the characteristic ions of the aromatic lactone, hence identified as tinocordifolin. Molecular formula for the parent ion [M + H]^+^ at *m/z* 521 was calculated as C_26_H_33_O_11_ with a difference of 13.93 ppm. Initial neutral loss of 162 Da generated the minor ion peak at *m/z* 359 that generated ion at *m/z* 313 due to loss of HCO_2_. Further fragments at *m/z* 205, 163, 131 and 121 indicates its lactone nature and columbin, hence structure was established as columbin glycoside; also known as palmatoside C. Precursor ion [M + H]^+^ at *m/z* 391 yielded the product ions at *m/z* 373 [M -H_2_O]^+^ and at *m/z* 355, 341, 327, 313 due to loss of H_2_O, CH_3_OH, CO and CH_3_COOH, 259, 121 and 107. It was tentatively identified as salvinorin B when compared with reference standard.

Low-energy CID-MS/MS of the protonated molecule [M + H]^+^ at *m/z* 307 afforded the following products ion at *m/z* 289, 177, 169, 153, 139 and 121 and identified as (-)-gallocatechin when compared with reference standard. Precursor ion [M + H]^+^ at *m/z* 315 yielded product ions at *m/z* 299 [M –CH_4_]^+^, 269 [M -H_2_C=O]^+^, 175, 145, 123, 107 were in accordance to methoxy-5,3′,4′-trihydroxyflavone. Precursor ion [M + H]^+^ at *m/z* 373 resolved at 28.4 min afforded product ions at *m/z* 357 [M –CH_4_]^+^, 341 [M –CH_4_ -CH_3_OH]^+^, 329, 317, 287, 205, 167, 137 was identified as pentamethoxyflavone. Protonated molecule [M + H]^+^ resolved at 23.9 min and at *m/z* 1031 yielded product ions at *m/z* 869 [M-Glu], 851, 743, 725 [M -Glu -Rhn], 705, 581 [M -Glu –Rhn -Rhn], 527, 415 [M -Glu –Rhn –Rhn -Glu]. Other product ions at *m/z* 397, 301, 273, 253 were comparable with diosgenin, hence identified as diosgenin 3-[glucosyl-(1- > 4)-rhamnosyl-(1->4)-[rhamnosyl-(1->2)]-glucoside. Parent ion [M + H]^+^ at *m/z* 415 resolved at 35.7 min yielded typical product ion at 135, 119, 109 similar to those lycopene, exact identification could not be done, hence named as lycopene derivative.

Precursor ion [M + H]^+^ at *m/z* 230 was identified as amino-tridecanoic acid as it yielded ions at *m/z* 212 [M –H_2_O]^+^, 195 [M –H_2_O –NH_3_]^+^, 184 [*m/z* 212 –CO]^+^, 167, 155, 141, 127 and 113 comparable to reference spectra. The pronated peak [M + H]^+^ at *m/z* 313 was identified as 3-oxo-nonadecanoic acid, as it yielded product ions at *m/z* 295, 277, 267, 253, 225, 211, 169, 141, 127 and 113 in same fashion as amino-tridecanoic acid. Precursor ion [M + H]^+^ resolved at 27.2 min of *m/z* 555 yielded products ions at *m/z* 359, 327, 309, 299, 281, 265, 247, 229, 215, 181, 177, 159, 139, and 124. Product ions at *m/z* 327 to 153 are the exact match with tetrahydroxy-octadecenoic acid; further initial loss of 196 Da indicated the loss of 2H_3_PO_4_ molecules. Hence, it was identified as phospholipid. Precursor ion [M + H]^+^ at *m/z* 387 could not be identified. However, the product ions at *m/z* 371, 353, 311, 290, 268, 203, 177, 175, 161, 147, 149, 135, 121 confirmed its steroidal nature. Another pronated molecule [M + H]^+^ at *m/z* 397 was identified as stigmastan-3,5-diene by comparing with reference standard as it yielded product ions at *m/z* 381, 301, 287, 285, 247, 229, 189, 175, 147, 135, 125, 109. Precursor ion [M + H]^+^ at *m/z* 495 resolved at 26.5 yielded products ions at *m/z* 441, 423, 371, 357, 339, 357, 339, 329, 311, 301, 283, 233, 219, 179, 145, 113. It was tentatively assigned as auricularine like compound by matching the fragmentation pattern with standard spectra. Phosphatidylcholine, choline, robinobiose, and glycosminine were identified using reference spectrum of these compounds.

### Quantitative analysis of bioactive compounds

The developed method was used to quantify 5 major metabolites in 21 samples of *T*. *cordifolia* grown on different host trees. The selective ion monitoring mode in Q-TOFMS was used to monitor selective and specific ions fragmentation pattern of 5 compounds. Quantitative Mass Hunter (B.04.00) was used to develop calibration curves of standards at different concentrations. The correlation coefficient was determined by using a linear regression model. The limits of detection (LOD) and limits of quantification (LOQ) were measured with a signal-to noise ratio (S/N) of about 3 and 10. The correlation coefficients r^2^ (0.9784–1.000), LOD (0.18–4.25 ng), and LOQ (6.03–15.63 ng) were observed for five standards used in the study (Table 4). Calibration curve for the standard compounds exhibited good linearity in the measured range of 0.25–50 ng/mL. The coefficients of linear regression were determined from 5 independent experiments. The calibration curves by means of weighted (1/x2) least squares linear regression were y = 0.24x + 0.026 and r^2^ = 0.9892 for berberine, y = 0.029x − 0.0025 and r^2^ = 0.9987 for jatrorrhizine, y = 0.084x + 0.027 and r^2^ = 0.9992 for palmatine, y = 0.122x + 0.028 and r^2^ > 1.000 for choline and y = 0.0962x + 0.343 and r^2^ = 0.9984 for magnoflorine. On the basis of calibration curves, the contents of berberine, choline, palmatine, magnoflorine, and jatrorrhizine in *T*. *cordifolia* extracts were quantified (Table 5). AIN group was found to have highest content of jatrorrhizine (2.88 ng/mL) as noticed in the qualitative analysis. FBG group was found to be endowed with significantly high concentrations of magnoflorine and choline confirmed the validity of qualitative analysis. Choline, a major component of cell wall was found to be highest in control (*T*. *cordifolia* climbed on the steel pole). ALC was found to have highest levels of palmatine (4.16 ng/mL) (Table 5). Overall, quantitative analysis confirmed the authenticity of results obtained in qualitative analysis.

**Table 4:** Test range, linearity, retention time, regression, slope, LOD, and LOQ for the reference compounds.

S. No.	Compounds	Test range (ng/ml)	Linear range (ng/ml)	Rt (min)	R^2^	Slope	LOD (ng)	LOQ (ng)
1.	Berberine	1.2–1200	1.9–200.6	30.09	0.9892	7.9482	1.98	5.971
2.	Palmatine	1.2–1200	2.9–201	32.30	0.9992	7.9454	1.60	4.994
3.	Jatrorrhizine	0.4–1200	2.1–199	28.77	0.9987	8.0049	4.25	12.87
4.	Magnoflorine	0.6–1200	2.7–201	28.35	0.9984	7.9272	2.24	6.80
5.	Choline	2.0–1200	4.6–200.8	7.04	1.0000	7.9847	0.18	0.5289

**Table 5:** The concentrations of berberine, palmatine, jatrorrhizine, magnoflorine, and choline (ng/mL) in *T*. *cordifolia* climbed on different trees.

S. No.	Compounds	AIN	ALC	ALL	ANI	FBG	TMI	Control
1.	Berberine	0.06	0.02	0.02	0.21	0.01	0.01	0.01
2.	Palmatine	1.34	4.16	1.81	1.78	2.21	1.97	2.23
3.	Jatrorrhizine	2.88	0.11	0.04	ND	0.10	0.13	1.31
4.	Magnoflorine	5.93	7.65	1.53	0.76	6.73	5.76	7.76
5.	Choline	4.45	5.12	ND	1.12	6.10	6.02	6.47

## Discussion

To explore the chemoprofiles of *T*. *cordifolia* climbed on different trees, a simple but efficient HPLC-QTOF-MS based method was used. HPLC-QTOF-MS method is very precise having >98% interday and intra-day accuracy. It has also recovered > 98% of seck its efficiency (Table 1). Both factors are important to standardize in order to minimize the variability and error rate across the data, in particular for qualitative analysis. Retention time alignment and mass data intensity normalization also affect the quality of analysis. Hence, the minimum difference in retention time and mass error noticed in this study were <0.05 min and <5 ppm respectively that further enhanced the data quality. Multivariate analysis methods are strong tools to differentiate the variations across the data^21^. In this study SVM and NB classifiers have been found to be the most accurate with r^2^ < 0.97. To decrease the false discovery rate, SNK post-hoc test with permutative (n = 50) Bonferroni FDR multiple testing correction was employed to increase the confidence in the analysis. The Venn diagram analysis has predicted the unique or the most abundant compounds across the different groups that can further be used as markers to identify the specific groups (Table 2).

*T*. *cordifolia* co-occurred with *A*. *indica* (AIN) contains 8-hydroxytinosporide and one unknown triterpenoid that can be used to differentiate this group from others. 5-Allyloxysalvigenin, trans-farnesol, reticuline and N-isovaleroylglycine are found in the control (*T*. *cordifolia* co-occuredwith steel pole), whereas, palmatoside C, D-glucan and 5-aminovaleric acid were found only in the ANI (*T*. *cordifolia* co-occurred with *A*. *nilotica*). Other group specific compounds include tinocordifolioside, tinoridine, tinosporinone, baenzigeroside A, tinosinen and 11-hydroxymustakone. All these compounds are found to be the group specific. However, medicinal importance of these compounds needs to be explored. Further fold change analysis have shown the highest variables in the *T*. *cordifolia* that co-occured with *A*. *indica* and steel pole or AIN and CON groups. Least number of variables are observed in TMI, ALL and FBG groups, i.e. *T*. *cordifolia* co-occured with *T*. *indica*, *A*. *lebbeck* and *F*. *benghalensis* respectively (Table 3). These qualitative results are further verified by quantitative analysis with some standard compounds. It is noticed that AIN contains the highest quantity of jatrorrhizine (2.88 ng/mL) and ALC group contains palmatine (4.16 ng/mL) and magnoflorine (7.65 ng/mL) (Table 5). The results of quantitative analysis are in accordance with qualitative analysis and have increased the confidence in the data. AIN group is also found to be a rich source of specific and up-regulated terpenoids including 8-hydroxytinosporide, unidentified terpenoids of 457 Da, borapetoside D, N-methylcoclaurine, isotanshinone II, oblongine, tinosporaside and cycloeucalenol, alkaloid jatrorrhizine and flavanoids i.e. peonidin, 5-allyloxysalvigenin and chrysin (Table 4). More specifically, AIN have increased contents of terpenoids along with specific alkaloid that synthesized from different route and fllvanoids.

Plants are chemically divergent and differe in biochemicals with plant species^22–26^. The plant chemistry mediates ecological interactions and influence the selection traits including specific constitutes^27^. Inter and intra-specific ecological interactions or competition within plant communities has been reported to influence the selection of an allelopathic secondary plant compound^28^. Hence, various kinds of selection forces may lead to different chemotypes within a plant species. In the current study, AIN group which is reported with the highest immunomodulatory activity is found to have high levels of terpenoids, jatrorhizine, flavonoids, coumarins, phytosterols and other categories compounds. The 8-hydroxytinosporide, scalar-17(25)-en-19-ol, borapetosides D, cycloartane-24,25-diol-3-one, palmarin, tinosporaside, and isotanshinone IIA are the terpenoids. One study has reported a significant decrease in concentrations of tinosporaside and berberine during winter season^29^. However, in the group AIN tinosporoside is found to be elevated as compared to other groups. High levels of borapetoside D, isotanshinone II, cycloeucalenol, chrysin, and methylcoumarin are found in AIN. These metabolites have been reported to have antioxidant properties and increase glucose uptake^30–34^. Tanshinone II, an analogue of isotanshinone II has been reported to have anti-fatigue properties by increasing the muscle glucose uptake and decreasing the lactic acid production^35,36^. Tanshinone II A is also found to be effective in treatment of cardiac problems despite of its less absorption through intestine^37^.

Concentrations of isoquinoline alkaloids i.e. N-methylcoclaurine, isoquinolone alkaloid, jatrorrhizine, oblongine, and haplopine are also found to be the highest in AIN group. N-methylcoclaurine inhibits the solute carrier organic anion transporter family member 1B1 and 1B3^38^. It has also been established that jatrorrhizine, oblongine, N-methylcoclaurine and magnoflorine are agonist of dopamine receptors^39,40^. Compounds that are monoamine depletors, could be useful to treat Parkinson or Huntington’s disease. Interaction of these alkaloids with dopamine receptors also modulate c-AMP metabolic process and cytosolic calcium ion concentration that are important in regulation of energy balance and other metabolic processes^41–43^. These alkaloids being dopamine receptors agonists activate trigeminal motoneurons that increase the muscle tone^44^. These compounds along-with above reported terpenoids, flavonoids and coumarin are also capable to modulate MAPK, ERK1, ERK2 and protein kinase A that are involved in signal transduction pathways to control the innate and adaptive immunity and cell death^45–47^. Cinnamaldehyde and sodium thiosalicylate along-with 20-hydroxyecdysone elevated in AIN group are known anti-inflammatory compounds and boost up immune system^48–50^. Hence, synergistic effect of these compounds along with jatrorrhizine might be a possible reason of the highest immunodulatory activities of the crude extract of *T*. *cordifolia* co-occured with *A*. *indica*. However, the role of other compounds present in minute quantities cannot be ignored and need to studied carefully.

The present study has clearly established that different chemotypes of *T*. *cordifolia* are due to biotic interactions with other plants. Levels of choline contents is affected as observed in the present study by interspecific interactions and therefore, the cell membrane composition for the ease of working of interactive compounds from both species. Hence, *T*. *cordifolia* co-occured with *A*. *indica* have the best medicinal efficacy due to the presence of high concentrations of specific terpenoids, alkaloids, and flavonoids which affect ERK1, ERK2 and MAPK cascade, c-AMP metabolic process, cytosolic calcium ion concentration and angiogenesis. However, more studies are required to explore the individual or syngesitic mechanism of these compounds. The study also emphasizes the necessity to study secondary metabolites accumulation in the plants under normal and biotic interaction conditions. Identification and understanding of the biotic and abiotic factors which influence the secondary metabolites production in *T*. *cordifolia* may also help to increase its medicinal efficacy. Better understanding of the ecological interactions due to co-occurrence of *T*. *cordifolia* with other plants will help to increase its medicinal efficacy and also to understand physiology of plant.

## Methods

### Standard compounds and chemicals

Standard compounds, berberine, jatrorrhizine, palmatine, magnoflorine, choline, ecdysteroids, lidocaine, D-camphor and 5,7-isoflavone were purchased from the Sigma-Aldrich, St. Louis, MO, USA. Acetonitrile, formic acid and water of LC-MS grade were purchased from the Sigma-Aldrich.

### *T*. *cordifolia* samples

Female *T*. *cordifolia* samples (protected from the use of any type of pesticides and organic material) were collected from a botanical garden, NRIBAS, Pune (with GPS location coordinates Latitude: 18.495°N, Longitute: 73.8053°E), in September 2011. The three stem samples from four-year-old *T*. *cordifolia* co-occured with steel pole (Control), *Azadirachta indica* (Meliaceae) [AIN], *Albizia lebbeck* (Fabaceae) [ALL], *Tamarindus indica* (Fabaceae) [TMI], *Ficus benghalensis* (Moraceae) [FBG], *Acacia nilotica* (Fabaceae) [ANI], and *Acacia leucophloea* (Fabaceae) [ALC] were collected. The samples were authenticated and specimen samples were deposited in the herbarium division of NRIBAS, Pune (Voucher No; 1995).

### Preparation of extracts

The stem samples were washed with tap water followed by deionized water to remove soil and other traces. These were dried in the air for 4 weeks. The air-dried stems were chopped and further converted into fine small pieces by mixer grinder (Philips, India). Dried stems (10 g) was extracted overnight with deionized water (Direct-Q, Millipore) (1:1 w/v) in orbital shaker at 37 °C and 180 rpm to yield the thick juice. Extracts were then centrifuged at 15,000 g for 10 min at 4 °C. The extraction was repeated three times for each sample and the supernatant was collected. The percolate from three repeats of each sample was then concentrated in a rotary vacuum evaporator at 50–60 °C. The supernatant juice was quick-freezed at −80 °C (Thermo-Fisher, Germany) and lyophilized (Freezone 4.5 Labconco, CA, USA) to yield a dry homogenous powder (0.3 g) and stored at −80 °C. The lyophilized powder from various samples was reconstituted in LC-MS grade water to make a solution of 5.0 mg/ml. Solutions were vortexed and centrifuged at 15,000 g for 20 minutes at 4 °C temperature. The supernatants were carefully removed and filtered through 0.22 µm syringe filters and transferred to 96 well plates. The complete workflow of the study design is depected in Fig. S1.

### HPLC-ESI-QTOF-MS

*T*. *cordifolia* stem extracts were resolved over ZORBAX Eclipse Plus reversed phase column (C18, 2.1 × 250 mm) having particle size 5 µm. Auto-sampler and column temperatures were maintained at 4 °C and 40 °C. Injection volume was kept constant, i.e. 20 µl for all samples. Chromatographic separation was carried out with Agilent 1200 Series HPLC interfaced to an Agilent 6520 Accurate-Mass QTOF-MS, with mobile phase A (water containing 0.1% formic acid) and B (acetonitrile containing 0.1% formic acid). The gradient program was carried as follows: 0–8 min, 5% B; 9–15 min, 5–20% B; 16–22 min, 20–45% B; 23–30 min, 45–65% B; 31–35 min, 65–90% B; 36–40 min, 90–5% B; and 41–45 min, 5% B. Flow rate of 0.5 ml/min was kept constant. Q-TOF-MS was operated in a positive ion polarity mode and extended dynamic range (1700 *m/z*, 2 GHz) with following parameters: gas temperature 350 °C, nebulizer 50 Psi, gas flow 11 L/Min, capillary voltage 3500 V, fragmentor voltage 175 V, nozzle 500 V, skimmer voltage 65 V, octapole RF 250 V and octapole DC1 48 V. Accurate MS/MS spectra were acquired in the range 100–1100 *m/z* with acquisition rate 3 spectra s^−1^. To assure the mass accuracy of recorded data, standards of lidocaine (234.3 *m/z*) and 5,7-isoflavone (284.3 *m/z*) were infused with samples along with continuous internal calibration. Overlay TIC of all the extracts depicting changes in the metabolites between various groups is represented in Fig. S2.

### Quality assurance

Standard compound mixture of 5 ppm was injected to check the operational conditions of the MS before sample analysis. Analogue compounds of standards in samples were identified on the basis of their retention time and the product ions. Quality control of extracts was also performed by mixing the three extracts from individual groups so that actual levels of metabolites can be compared with the individual sample of that group. Another quality control check was kept from mixing the extracts with standards (2.5 ppm) to check the peak volumes and intensity. After identification, comparative concentration of the analytes was calculated by its area and intensity ratio.

### Multivariate statistical analysis

MassHunter (Qualitative Analysis Version B.04.00) and Profinder (Version B.06.00) softwares (Agilent Technologies) were used to process raw mass data. Molecular features presents in raw data sets were extracted keeping minimum ion abundance 5000 cycles per sec (cps), peak space tolerance 0.0025 *m/z* or 5 ppm and relative height 2.5% (Fig. S3). Processed data sets were imported into the Mass Profiler Professional (MPP) software (Version.B.02.02; Agilent Technologies) and aligned using retention time window and mass difference 0.2 min and 5.0 ppm. A molecular feature present in less than 3 samples of a group and having p > 0.05, fold change ≤ 2.0 and CV > 15 were removed from datasets. Refined data were further subjected to multivariate analysis to prepare five different discrimination models, namely partial least square discrimination analysis (PLS-DA), Support Vector Machines (SVMs), Naive Bayes (NB), Decision Tree (DT), and Neural network (NN). All the models were trained and used for further identification of unknown samples. Discriminated data sets from best model were subjected to statistical analysis using One-way ANOVA Student-Newman-Keuls (SNK) post-hoc test with Bonferroni false discovery rate (FDR) multiple testing correction and asymptotic p values were computed. Principal component analysis (PCA) was performed using the molecular feature (metabolites) having p < 0.001 and fold change > 2.0 in at least one group. Finally, all the metabolites having any missing value were removed.

### Quantitation of selected compounds using Selected Ion Monitoring (SIM)

All the standards and extracts were subjected to selected ion monitoring with defined collision energies. For the quantification the transitions at *m/z* 342.16 → 297.03 for magnoflorine, *m/z* 336 → 320 for berberine, at *m/z* 352 → 336 for palmatine, at *m/z* 338 → 322 for jatrorrhizine, and at *m/z* 146.13 → 86.93 for choline were monitored in SIM mode of Q-TOF-MS keeping scan time 0.3 s per transition. Varying concentrations of standards were used to construct standard curves and metabolites in the extracts were quantified by using Agilent MassHunter Quantitative Analysis Software (Version B.02.00). A window of 100 ppm was set for fragment identification. Standards and their corresponding metabolites in the extracts were quantified using the peak size of the fragment by extracting ion chromatogram function.

## Supplementary information

Supplementary Information
