# Supplementary material for: Interactions of a medicinal climber Tinospora cordifolia with supportive interspecific plants trigger the modulation in its secondary metabolic profiles
Source: Sci Rep. 2019 Oct 4;9:14327. doi: 10.1038/s41598-019-50801-0 (PMC6778175; doi:10.1038/s41598-019-50801-0)
Supplement: Supplementary file 1 — Supplementary Information [file 41598_2019_50801_MOESM1_ESM.pdf]

Interactions of a medicinal climber *Tinospora cordifolia* with supportive interspecific plants trigger the modulation in its secondary metabolic profiles

Bhawana Sharma, Aarti Yadav, Rajesh Dabur*

Department of Biochemistry, Maharishi Dayanand University, Rohtak, Haryana, India-124001

*Corresponding Author
Rajesh Dabur
Head, Department of Biochemistry,
Maharishi Dayanand University,
Rohtak, Haryana-124001
E-Mail: rajeshdabur@yahoo.com
Phone: +911262393070

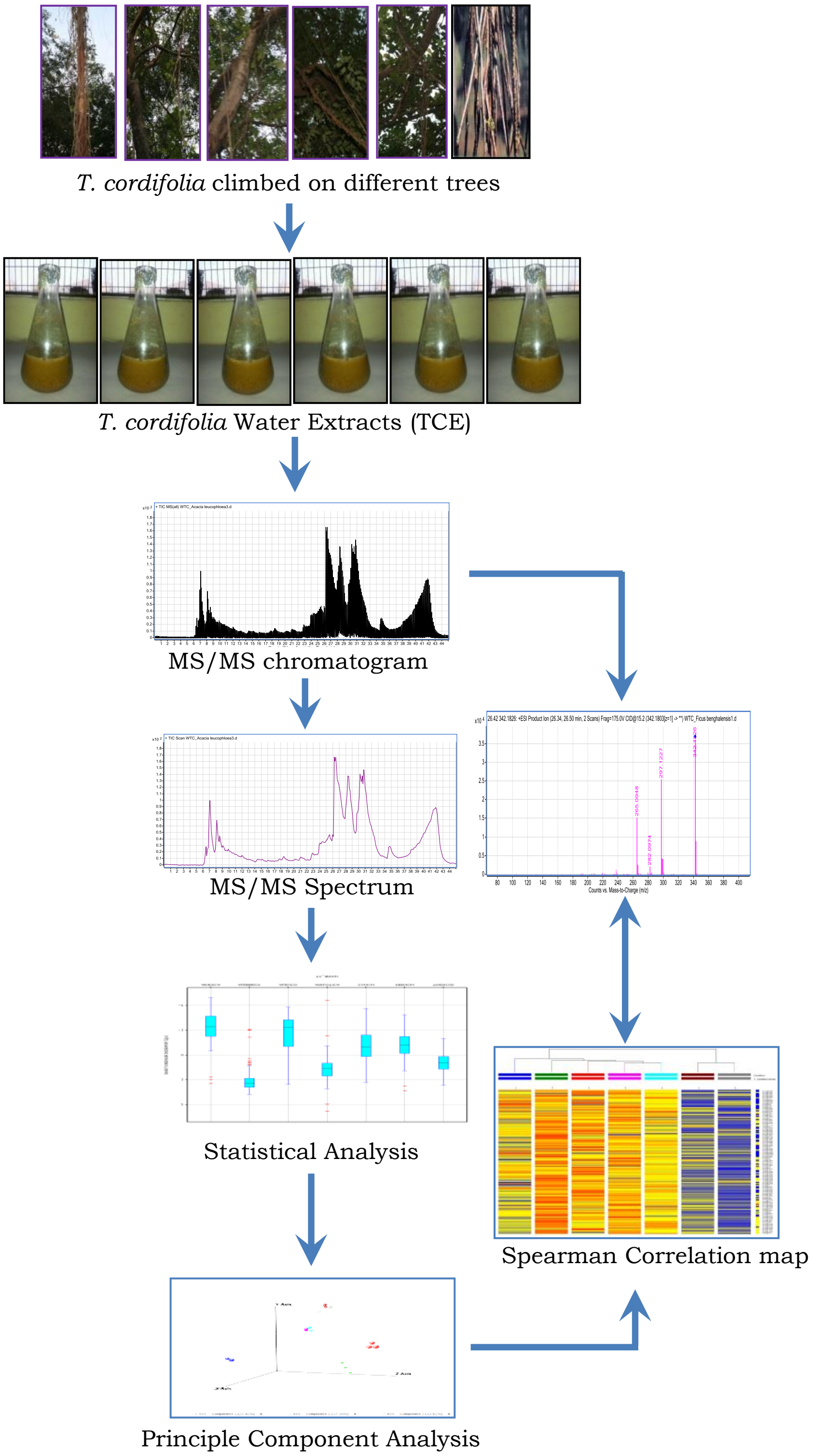

Figure S1: Figure is showing flow diagram of study. From different samples of *T. cordifolia* water extracts were prepared and subjected HPLC-Q-TOF-MS to collect MS/MS data. MS/MS data was extracted for MS data and statistical analyses were performed. Differential metabolites in different samples of *T. cordifolia* were identified by comparing MS/MS finger prints from data to standard compounds and libraries.

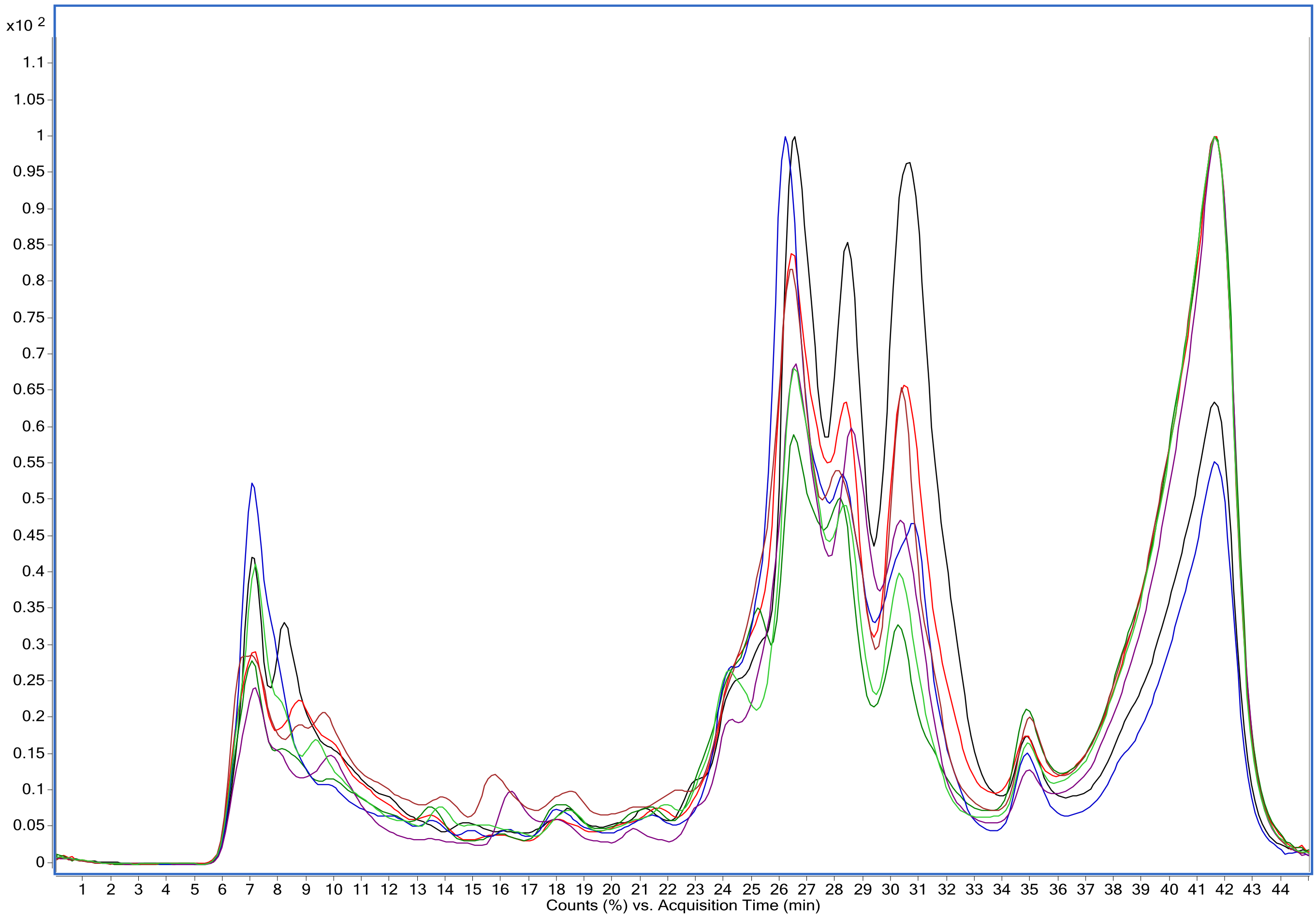

Figure S2: Figure is showing overlay TIC of all the extracts to understand the changes in the metabolites of various groups i.e. CON (violet), ALC (black), ANI (red), ALL (green), AIN (blue), FBG (purple).

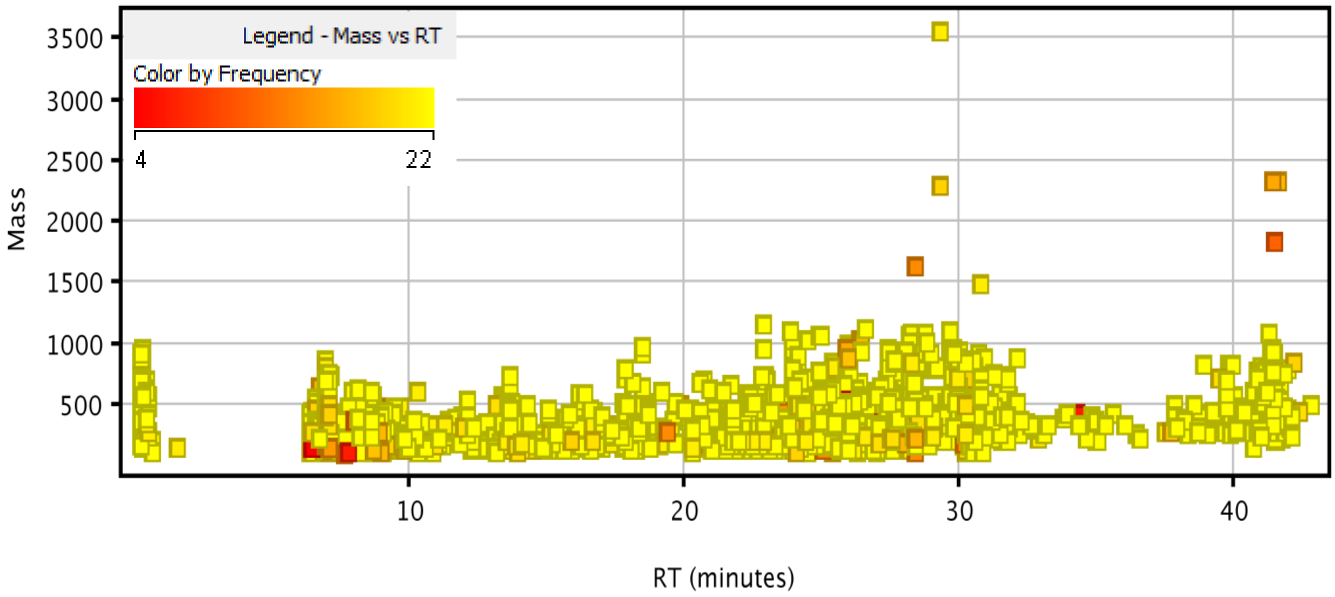

Figure S3: Molecular features present across the sample after retention time and *m/z* alignment. Colour scale from yellow to red showing frequency of molecular features present in different samples.

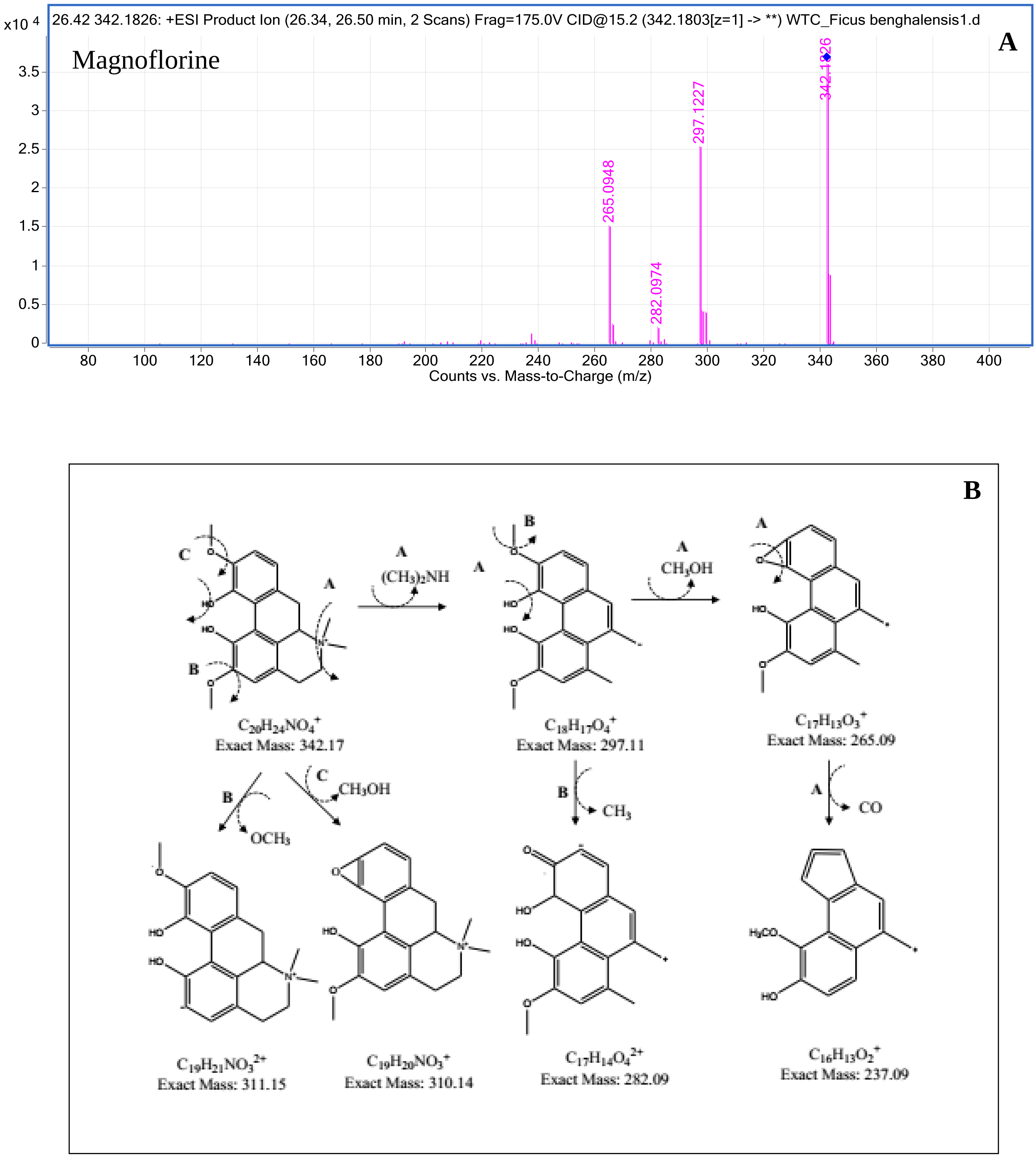

x10 4
26.42 342.1826: +ESI Product Ion (26.34, 26.50 min, 2 Scans) Frag=175.0V CID@15.2 (342.1803[z=1] -> **) WTC_Ficus benghalensis1.d
A
Magnoflorine
342.1826
297.1227
265.0948
282.0974
Counts vs. Mass-to-Charge (m/z)
B
(CH3)2NH
CH3OH
OCH3
CH3
CO
C20H24NO4+
Exact Mass: 342.17
C18H17O4+
Exact Mass: 297.11
C17H13O3+
Exact Mass: 265.09
C19H21NO3 2+
Exact Mass: 311.15
C19H20NO3+
Exact Mass: 310.14
C17H14O4 2+
Exact Mass: 282.09
C16H13O2+
Exact Mass: 237.09

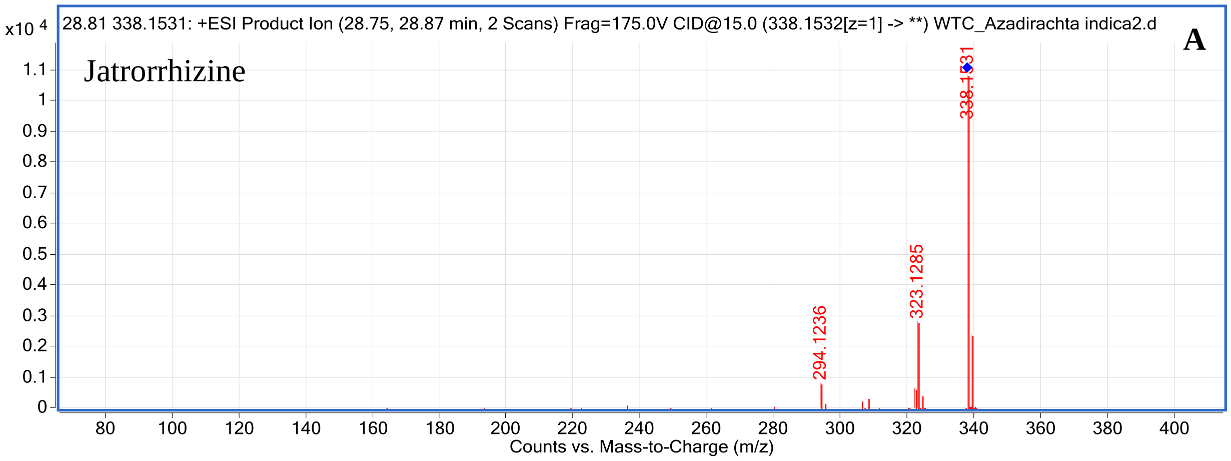
x10 4
28.81 338.1531: +ESI Product Ion (28.75, 28.87 min, 2 Scans) Frag=175.0V CID@15.0 (338.1532[z=1] -> **) WTC_Azadirachta indica2.d
A
Jatrorrhizine
338.1531
323.1285
294.1236
Counts vs. Mass-to-Charge (m/z)

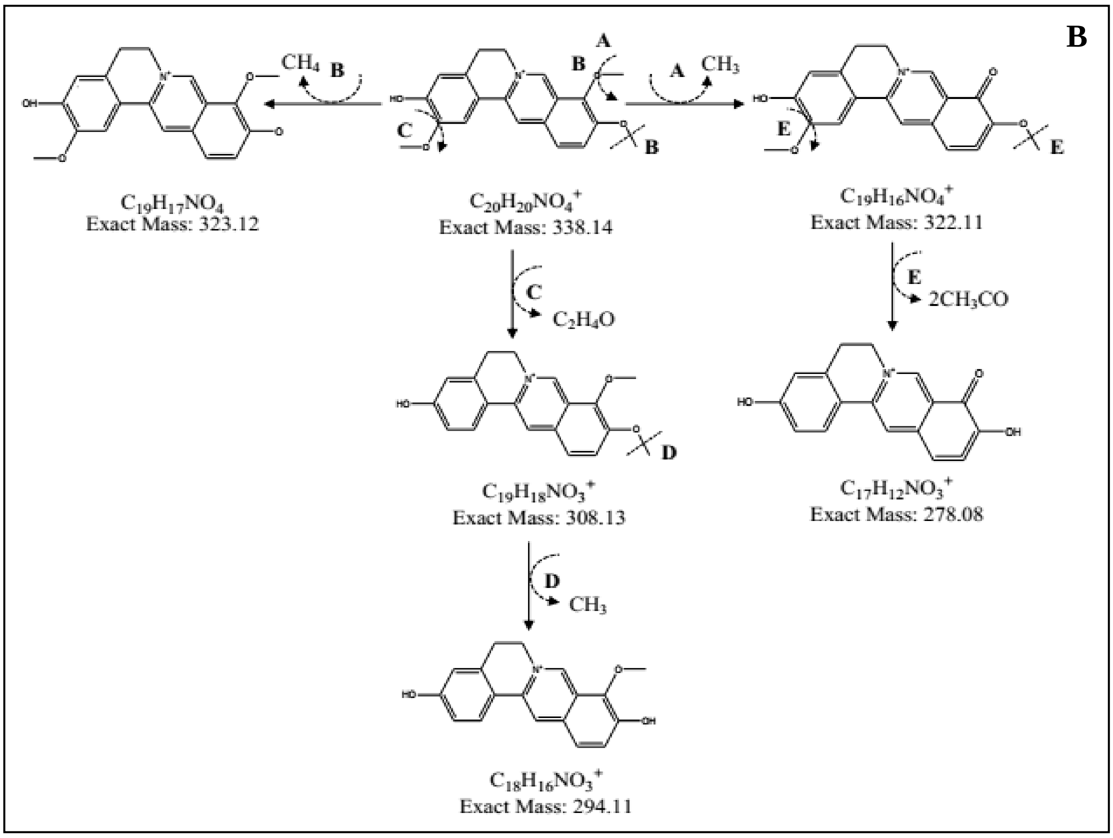
B
CH4 B
A CH3
C
E
C19H17NO4
Exact Mass: 323.12
C20H20NO4+
Exact Mass: 338.14
C19H16NO4+
Exact Mass: 322.11
C C2H4O
E 2CH3CO
C19H18NO3+
Exact Mass: 308.13
C17H12NO3+
Exact Mass: 278.08
D CH3
C18H16NO3+
Exact Mass: 294.11

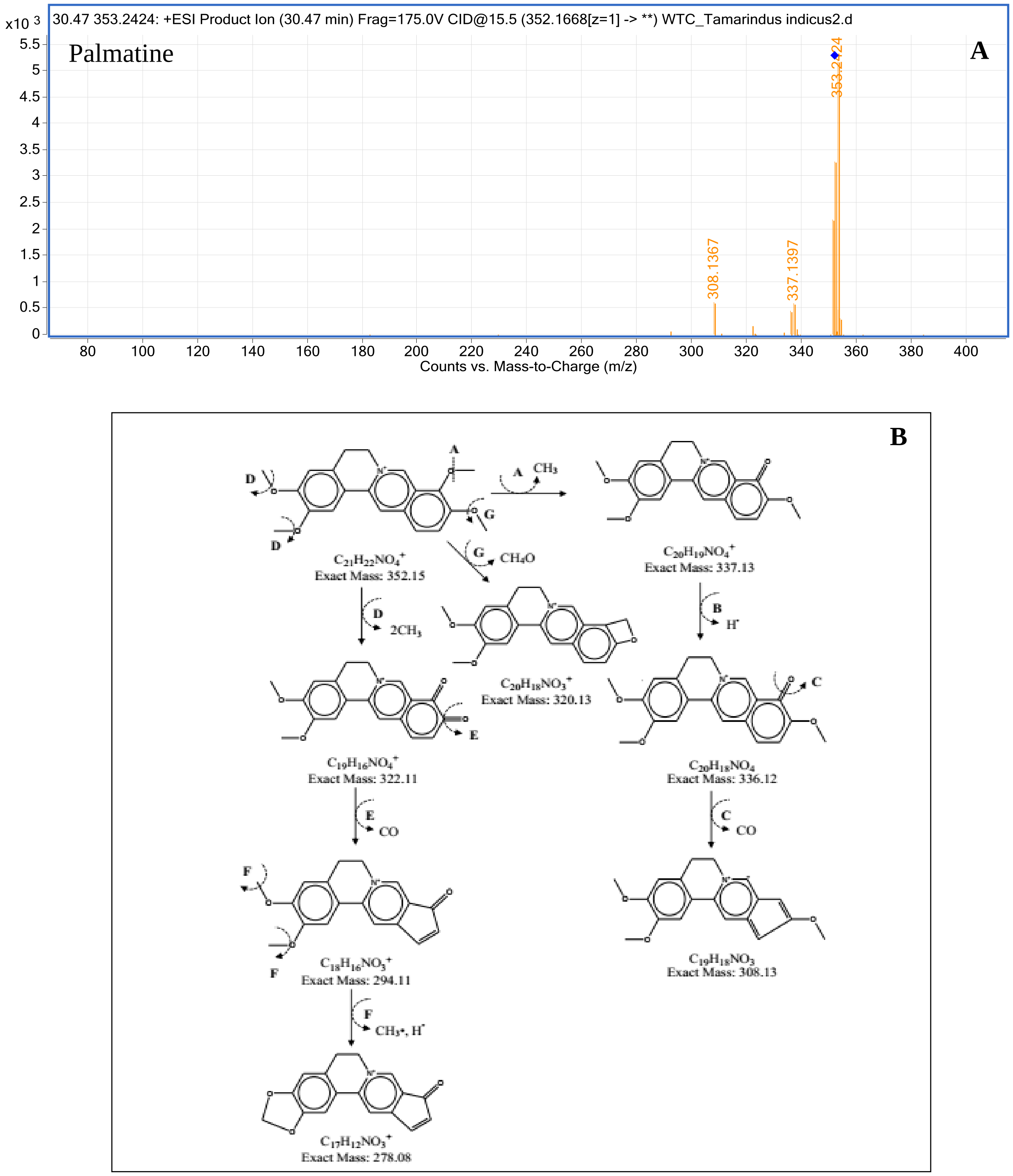

Figure S4: MS/MS spectra **(A)** and fragmentation pattern **(B)** of some important metabolites from *T. cordifolia*.

Table S1: Table is showing overall statistical data after analysis.

Property	[AIN]	[ALC]	[ALL]	[ANI]	[CON]	[FBG]	[TMI]
No. of Observations	136	136	136	136	136	136	136
No. of Missing Values	0	0	0	0	0	0	0
Minimum	-1.388	-2.08973	-1.99892	-2.14862	-1.35003	-2.05256	-1.52561
Maximum	2.177304	2.078623	0.749191	0.819054	1.846073	2.013044	1.535707
Mean	0.52304	-0.09948	-0.92466	-1.20491	0.797094	0.49885	0.14437
Trimmed Mean	0.524887	-0.10084	-0.92897	-1.21268	0.804995	0.506312	0.146375
Median	0.542959	-0.13001	-0.98942	-1.33528	0.877361	0.591215	0.200295
Std. Deviation	0.712546	0.801875	0.591698	0.582981	0.610609	0.644576	0.525342
Trimmed Std. Deviation	0.684677	0.767619	0.571399	0.555512	0.580348	0.598045	0.495725
No. Of Outliers	2	0	5	8	3	8	7
Percentile 1.0	-1.3861	-2.07543	-1.98589	-2.1179	-1.21923	-1.95434	-1.42183
Percentile 5.0	-0.80185	-1.67025	-1.86667	-1.90898	-0.44658	-0.69844	-0.89617
Percentile 10.0	-0.45367	-1.02338	-1.62998	-1.7793	-0.05066	-0.30709	-0.55961
Percentile 25.0	0.096197	-0.66606	-1.30601	-1.60525	0.462673	0.234511	-0.10406
Percentile 50.0	0.542959	-0.13001	-0.98942	-1.33528	0.877361	0.591215	0.200295
Percentile 75.0	1.046418	0.516958	-0.63603	-0.94794	1.249414	0.86813	0.479283
Percentile 90.0	1.423768	0.893942	-0.05842	-0.3563	1.503839	1.201966	0.797126
Percentile 95.0	1.522178	1.134242	0.206066	0.242356	1.586965	1.516074	0.939623
Percentile 99.0	2.045693	1.966692	0.720571	0.650602	1.82837	1.91128	1.492031

Table S2: Discrimination ability of different trained models analysed in the present study.

Identifier	TC Winter	Partial Least Squares Discrimination (Predicted)	Partial Least Squares Discrimination (Confidence)	Support Vector Machine (Predicted)	Support Vector Machine (Confidence Measure)	Naive Bayes (Predicted)	Naive Bayes(Confidence Measure)	Decision Tree (Predicted)	Decision Tree (Confidence Measure)	Neural Network (Predicted)	Neural Network (Confidence Measure)
AIN: Log2	[AIN]	[AIN]	0.8009307	[AIN]	1	[AIN]	1	[CON]	1	[AIN]	1
AIN2: Log2	[AIN]	[AIN]	0.9387083	[AIN]	1	[AIN]	1	[TMI]	1	[AIN]	1
AIN3: Log2	[AIN]	[AIN]	0.823077	[AIN]	1	[AIN]	1	[ALC]	1	[ALL]	1
ALL1: Log2	[ALL]	[ALL]	0.92909354	[ALL]	1	[ALL]	1	[ALL]	1	[ALL]	1
ALL1_1: Log2	[ALL]	[ALL]	0.82878554	[ALL]	1	[ALL]	1	[ALL]	1	[ALL]	1
ALL2: Log2	[ALL]	[ALL]	1	[ALL]	1	[ALL]	1	[ALL]	1	[ALL]	1
ALL2_1: Log2	[ALL]	[ALL]	0.835974	[ALL]	1	[ALL]	1	[ALL]	1	[ALL]	1
ALL3: Log2	[ALL]	[ALL]	0.82899284	[ALL]	1	[ALL]	1	[ALL]	1	[ALL]	1
ALL3_1: Log2	[ALL]	[ALL]	0.8302213	[ALL]	1	[ALL]	1	[ALL]	1	[ALL]	1
ALCa1: Log2	[ALC]	[ALC]	0.8800626	[ALC]	1	[ALC]	1	[ALC]	1	[AIN]	1
ALC2: Log2	[ALC]	[ALC]	0.7886802	[ALC]	1	[ALC]	1	[ALC]	1	[ALC]	1
ALC3: Log2	[ALC]	[ALC]	0.8711175	[ALC]	1	[ALC]	1	[ALC]	1	[ALC]	1
ANI1: Log2	[ANI]	[ALL]	0.59169155	[ANI]	1	[ANI]	1	[ALL]	1	[CON]	1
ANI2: Log2	[ANI]	[ALL]	0.60480255	[ANI]	1	[ANI]	1	[ANI]	1	[ANI]	1
ANI3: Log2	[ANI]	[ALL]	0.7111883	[ANI]	1	[ANI]	1	[ANI]	1	[ANI]	1
CON1: Log2	[CON]	[CON]	0.88335756	[CON]	1	[CON]	1	[CON]	1	[CON]	1
CON2: Log2	[CON]	[CON]	0.8721478	[CON]	1	[AIN]	1	[CON]	1	[CON]	1
CON3: Log2	[CON]	[CON]	0.76880735	[CON]	1	[CON]	1	[CON]	1	[CON]	1
CON4_1: Log2	[CON]	[CON]	0.9604613	[CON]	1	[CON]	1	[CON]	1	[CON]	1
FBG1: Log2	[FBG]	[FBG]	0.77363956	[FBG]	1	[FBG]	1	[FBG]	1	[AIN]	1
FBG2: Log2	[FBG]	[FBG]	0.77097815	[FBG]	1	[FBG]	1	[FBG]	1	[FBG]	1
FBG3: Log2	[FBG]	[FBG]	0.73211	[FBG]	1	[FBG]	1	[CON]	1	[FBG]	1
TMI1: Log2	[TMI]	[CON]	0.95754486	[TMI]	1	[TMI]	1	[FBG]	1	[TMI]	1
TMI2: Log2	[TMI]	[ALL]	0.87112653	[TMI]	1	[TMI]	1	[TMI]	1	[ALC]	1
TMI3: Log2	[TMI]	[ALL]	0.81691746	[TMI]	1	[TMI]	1	[CON]	1	[TMI]	1

* Red values indicate the incorrectly identified samples by the trained models.

Table S3: Table is showing significant metabolites in different groups in comparison to control [CON] group. Mass error (Δppm) values were not corrected with stochastic noise and internal standards. After correction all values are less than 5 ppm.

S. No.	Tentative Identification	Compound	RT	Formula	Δppm	Fragments (*m/z*)
1.	Choline*	104.1070	7.08	$C_5H_{14}NO$	2.3	-
2.	Aminophenol*	109.0571	7.44	C_6H_7NO	11.3	-
3.	Betaine	117.0837	6.77	$C_5H_{11}NO_2$	8.7	101
4.	Phenylmethanethiol	124.0332	7.10	$C_5H_4N_2O_2$	21.9	107
5.	Cinnamaldehyde	132.0624	24.0	C_9H_8O	11.4	103, 115
6.	Isoquinoline N-oxide	145.0914	8.72	C_9H_7NO	12.2	104, 128
7.	6-Methylcoumarin	160.0583	23.9	$C_{10}H_8O_2$	3.5	103, 105, 115, 133
8.	Sodium thiosalicylate*	176.9952	7.10	$C_7H_5NaO_2S$	-	101, 113, 147, 159
9.	Acetamido-6-aminohexanoic acid	189.1617	27.9	$C_8H_{17}N_2O_3$	15.4	-
10.	1,3 Dimethylpteridine-2,4-dione	192.0856	24.0	$C_8H_8N_4O_2$	1.4	105, 115, 133, 161, 177
11.	Unknown	192.9735	7.08	$C_4H_5N_2O_3S_2^-$	7.2	103, 153, 175
12.	3-[4-(3-Aminopropylamino) butylamino]propanoicacid	217.2119	28.7	$C_{10}H_{23}N_3O_2$	7.6	145, 158, 187, 199 *(loss of 60 Da due to CH_3COOH formed peak at 158)*
13.	Ethyl ferulate	222.0645	41.4	$C_{12}H_{14}O_4$	14.4	139, 207
14.	Amino-tridecanoic acid*	229.2490	34.9	$C_{13}H_{27}NO_2$	7.9	212
15.	Glycosminine	236.0925	26.4	$C_{15}H_{12}N_2O$	12.55	135, 147, 161, 182, 194, 207, 222
16.	Haplopine*	245.2444	31.4	$C_{13}H_{11}NO_4$	19.6	106, 228
17.	Trimethyldecahydrophenanthren-2-ol	248.1147	25.8	$C_{17}H_{28}O$	0.1	141, 159, 187, 219, 231
18.	Tinocordifolin	250.1662	26.2	$C_{15}H_{22}O_3$	3.8	116, 128, 158, 175, 203, 222, 234
19.	Chrysin*	254.1615	33.1	$C_{15}H_{10}O_4$	12.1	103, 129, 153, 177, 209
20.	Palmitic amide	255.2656	34.8	$C_{16}H_{33}NO$	14.8	102, 116, 152, 196
21.	*Amyl p-butylaminobenzoate*	263.1830	31.7	$C_{16}H_{25}NO_2$	13.4	-
22.	Unknown	264.1830	26.4	-	-	-
23.	Neocryptotanshinone II	270.1381	28.2	$C_{17}H_{18}O_3$	8.9	-
24.	13-Methyl-17-norabieta-15-ene-8-ylium	273.2779	34.7	$C_{20}H_{33}$	4.5	257
25.	Unknown	278.1630	40.6	$C_{15}H_{22}N_2O_3$	0.2	-
26.	Alkaloid	281.0918	26.4	$C_{13}H_{15}NO_6$	7.3	107, 151, 206, 221, 236, 252, 267
27.	Carboxylic acid	281.1161	25.6	$C_{10}H_{19}NO_8$	17.5	147, 235
28.	Unknown	282.1359	28.2	$C_{14}H_{20}NO_5$	6.6	-
29.	Unknown	282.1783	23.6	C_{15} H_{26} N_2OS	5.0	-
30.	Coclaurine	285.1466	24.8	$C_{17}H_{19}NO_3$	29.9	107, 161, 255
31.	Magnoflorine-[(CH$_3$)2NH]	296.1156	26.4	$C_{18}H_{16}O_4^+$	3.8	166, 181, 207, 219, 237, 265, 282
32.	Unknown	298.1311	26.4	-	-	-
33.	N-Methylcoclaurine	299.1643	24.4	$C_{18}H_{21}NO_3$	7.2	107, 123, 135, 164, 192, 255, 287, 288
34.	Unknown ester (Floridimine type)	301.2001	24.1	$C_{15}H_{27}NO_5$	6.9	102, 255, 256
35.	Sphinganine	301.3094	38.1	$C_{18}H_{39}NO_2$	6.9	113, 127, 141, 155, 169, 183, 197, 252, 266, 284
36.	(-)-Gallocatechin	306.0222	8.03	$C_{15}H_{14}O_7$	6.4	129, 149, 189, 201, 217, 227
37.	3-Oxo-nonadecanoic acid	312.2785	41.4	$C_{19}H_{36}O_3$	14.6	117, 123, 137, 161, 200, 207, 215, 239, 257, 297
38.	Feruloyltyramine*	313.1428	25.4	$C_{18}H_{19}NO_4$	5.1	145, 177, 269, 299
39.	(+/-) Oblongine*	313.1793	27.0	$C_{19}H_{24}NO_3^+$	18.9	107, 237, 269, 281
40.	(+/-) Oblongine	313.1793	25.6	$C_{19}H_{24}NO_3^+$	3.8	107, 137, 175, 237, 254, 269
41.	Unknown	315.2159	25.8	$C_{21}H_{30}O_2$	30.5	-
42.	Phytosphingosine	317.3048	34.8	$C_{18}H_{39}NO_3$	26.2	102, 256, 300
43.	Robinobiose	326.1280	27.0	$C_{12}H_{22}O_{10}$	20.5	143, 172, 181, 195, 207,227, 265, 283, 291,

S. No.	Tentative Identification	Compound	RT	Formula	Δppm	Fragments (*m/z*)
						309
44.	Dideoxysulphonated steroid	326.2064	24.3	$C_{18}H_{30}O_3S$	25.8	109, 145, 169, 185, 199, 207, 219, 225, 247, 299
45.	Icosasphinganine	329.3416	41.0	$C_{20}H_{43}NO_2$	12.9	157, 215, 312
46.	Unknown	332.0168	7.20	$C_{14}H_{10}N_3O_3S_2$	1.7	-
47.	Jatrorrhizine*	337.1444	28.8	$C_{20}H_{20}NO_4^+$	14.9	294, 308, 322, 323
48.	Magnoflorine [M+]	342.1712	26.4	$C_{20}H_{24}NO_4^+$	1.4	219, 237, 265, 282, 297
49.	8-Oxoberberine	351.1594	29.8	$C_{20}H_{17}NO_5$	6.6	235,294, 308, 336, 337<br>The fragments formed due to [M+H–CH$_3$]+, [M+H–CH$_3$–H–CO]+, [M+H–CH$_3$–H$_2$O]+
50.	Palmatine*	352.2358	32.4	$C_{21}H_{22}NO_4^+$	0.3	292, 294, 308, 320, 322, 336, 337
51.	Corydine methyl ether	355.1914	27.4	$C_{21}H_{25}NO_4$	7.4	229, 236, 251, 264, 279, 296, 311
52.	N-Tetrahydropalmatine*	356.1393	29.0	$C_{21}H_{25}NO_4$	7.9	165, 192
53.	Isocorydine-N-oxide	357.1709	25.6	$C_{20}H_{23}NO_5$	6.6	285, 295, 313
54.	Isoquinolone alkaloid	357.2074	26.8	$C_{21}H_{27}NO_4$	13.9	137, 151, 177, 192, 313
55.	Tinosporin	358.1552	27.1	$C_{20}H_{22}O_6$	9.3	105, 143, 171, 189, 215, 247, 281, 295, 309, 327, 339
56.	Glucoside	368.1221	17.9	-	-	104, 207
57.	Unknown	369.3014	41.7	$C_{21}H_{39}NO_4$	8.3	105, 117, 133, 151, 175, 203, 227, 247, 267, 355
58.	Pentamethoxyflavone	372.1349	28.4	$C_{20}H_{20}O_7$	8.3	101, 125, 137, 155, 161, 197, 213, 235, 261, 269, 281, 299, 327, 355
59.	Palmarin	374.2572	41.8	$C_{20}H_{22}O_7$	6.5	150, 125, 165, 356, 357
60.	Steroidal Compound	387.2611	24.7	$C_{21}H_{39}O_6$	17.2	121, 133, 163, 177, 203, 247, 267, 291, 311, 353, 371
61.	Salvinorin B	387.2611	28.5	$C_{21}H_{26}O_7$	2.2	102, 123, 131, 143, 151, 173, 197, 215, 233, 267, 281, 299, 309, 327, 345, 355, 373
62.	Unknown	391.1773	29.8	$C_{25}H_{29}NO_3$	-	-
63.	Stigmastan-3,5-diene	396.3033	41.7	$C_{29}H_{48}$	6.6	105, 109, 125, 135, 147, 165, 175, 189, 203, 229, 243, 247, 285, 291, 297, 301, 381
64.	Unknown	401.2199	25.4	-	-	-
65.	Tinocordifolioside	412.2255	26.1	$C_{21}H_{32}O_8$	12.8	125, 147, 159, 175, 187, 197, 215, 233, 251
66.	Lycopene derivative	414.2205	35.7	-	13.9	109, 119, 135, 396
67.	Cycloeucalenol	426.2928	25.9	$C_{30}H_{50}O$	9.8	111, 145, 163, 175, 191, 209, 249, 287, 301, 331, 353, 371, 409
68.	Unknown	431.2893	25.0	-	-	-
69.	Unknown	435.4430	41.5	$C_{29}H_{57}NO$	2.3	113, 129, 135, 155, 197, 213, 231 283, 297, 311, 339, 355
70.	Unknown	440.3092	26.5	-	-	-
71.	Unknown	440.3313	41.7	-	-	-
72.	Glucoside	442.2119	24.5	-	-	147, 281
73.	3β,5α,14α-Trihydroxyergosta-7,22-dien-6-one	444.3046	25.9	$C_{28}H_{44}O_4$	22.4	123, 135, 165, 217, 247, 255, 301, 313, 331, 347, 371, 409, 427
74.	Glucoside of *m/z* 286	447.2062	23.4	-	-	107, 161, 255, 286
75.	Cycloartane-24,25-diol-3-one	458.3206	26.5	$C_{30}H_{50}O_3$	13.6	113, 137, 175, 199, 227, 301, 313, 329, 347, 371, 423, 441<br>Ions at *mlz* 313 and 175 represent characteristic fragments of 9,19-cycloartane-type triterpenes
76.	Unknown	467.9881	7.05	-	-	129, 240, 287, 315, 334, 428
77.	Unknown	468.1418	21.0	-	-	299, 317
78.	20-Hydroxyecdysone*	480.3273	25.9	$C_{27}H_{44}O_7$	3.5	109, 125, 165, 201, 235, 265, 283, 301, 331, 349, 371, 377, 391, 409, 427, 445, 463
79.	Unknown	483.9681	7.05	$C_{27}H_{22}N_3O_3S$	12.5	-
80.	Unknown	484.3696	25.0	$C_{30}H_{46}NO_4$	13.4	102, 184, 302, 385
81.	Auricularine	494.3421	26.5	$C_{33}H_{42}N_4$	2.12	109, 111, 139, 149, 179, 205, 219, 249, 263, 301, 311, 329, 339, 357, 371, 423, 441, 459
82.	Tinosporaside	500.1694	18.5	$C_{26}H_{28}O_{10}$	1.5	317
83.	Unknown	506.3421	41.6	$C_{33}H_{46}O_4$	4.7	-
84.	Baenzigeroside A	520.3468	25.2	$C_{26}H_{32}O_{11}$	17.4	103, 131, 163, 193, 313, 485
85.	Unknown	521.2311	23.9	$C_{29}H_{33}N_2O_7$	4.2	115, 133, 161, 193, 211

S. No.	Tentative Identification	Compound	RT	Formula	Δppm	Fragments (*m/z*)
86.	Unknown	523.1872	23.9	$C_{28}H_{29}NO_9$	7.8	115, 133, 161, 193, 211
87.	Unknown	526.1865	23.9	$C_{28}H_{30}O_{10}$	3.9	317
88.	Unknown	527.2571	13.4	$C_{33}H_{37}NO_5$	19.2	103, 121, 138, 201, 430, 467
89.	Unknown	539.2580	28.3	$C_{24}H_{37}N_5O_9$	2.0	171, 189, 203, 217, 243, 265, 297, 311, 325, 343
90.	Unknown	542.1611	23.9	-	-	129, 154, 201, 227, 284, 297, 363, 383, 431, 485
91.	O-Glucoside derivative	542.2257	28.0	-	-	363, 365
92.	Unknown	545.4140	41.6	-	-	-
93.	Unknown	555.2535	27.2	-	-	-
94.	O-Glucoside derivative	560.2090	25.2	-	-	203
95.	Isotanshinone II *	294.1231	30.2	$C_{19}H_{18}O_3$	8.8	109, 119, 125, 135, 147, 167, 179, 207, 257, 277
96.	Unknown	567.5813	41.2	-	-	123, 163, 239, 266, 284, 314, 368, 429, 467, 526, 552
97.	Unknown	572.4166	41.5	-	-	-
98.	Unknown	584.390	25.8	$C_{27}H_{40}O_{14}$	2.1	127, 179, 249, 283, 301, 353, 371, 391, 409, 427
99.	Unknown	589.4426	41.5	-	-	-
100.	Unknown	590.2206	25.7			183, 203, 429
101.	Cordifolide A	598.4068	26.9	$C_{28}H_{38}O_{14}$	-	103, 167, 193, 216, 247, 298, 385, 416
102.	Unknown	609.2013	41.4	-	-	221, 262, 281, 355, 401, 489
103.	Isoquinoline alkaloid	616.4461	41.5	-	-	161, 204, 218, 263, 337, 389
104.	Saponin glycoside	620.2330	25.6	-	-	-
105.	Chrysoeriol C-hexoside O-hexoside	624.2278	20.8			300, 351, 463
106.	Unknown cinnamic derivative	633.4716	41.4	-	-	-
107.	Unknown	652.3055	27.9	-	-	172, 413, 443, 471, 516, 608
108.	Glucoside of *m/z* 493	654.2404	21.7			367, 463
109.	Unknown	698.4278	27.4	$C_{33}H_{46}O_{16}$	4.5	301, 375, 537
110.	Unknown	701.2358	41.4	-	-	-
111.	Unknown	711.3952	28.1	-	-	-
112.	5-Allyloxysalvigenin derivative	714.2648	17.8	-	-	369
113.	Unknown	740.3128	30.4	-	-	269, 337, 383
114.	Unknown	748.3064	29.9	-	-	-
115.	Unknown	756.3090	30.4			269, 337, 353, 381, 397
116.	Unknown	759.6111	41.3	-	-	184
117.	Unknown	768.2930	27.6	-	-	-
118.	Unknown	785.4038	27.4	-	-	-
119.	Unknown of *m/z* 184.08	785.6281	41.2	-	-	100, 184
120.	Unknown	786.2847	29.9	-	-	353, 399, 413
121.	Unknown	786.3052	25.7	-	-	-
122.	Unknown	788.2965	29.8	-	-	-
123.	Unknown	902.7654	41.3	-	-	-
124.	Unknown	921.8029	41.3	-	-	-
125.	Unknown	928.7762	41.3	-	-	-
126.	Unknown	934.3666	28.5	-	-	545
127.	Unknown	940.4492	28.0	-	-	-
128.	Unknown	947.8217	41.3	-	-	100, 155, 277, 301, 467, 491, 533, 577, 615, 651, 669, 748, 815, 871, 889, 929
129.	Unknown	951.4175	23.9	-	-	269, 286, 448
130.	Diosgenin 3-[glucosyl-(1->4)-rhamnosyl-(1->4)-[rhamnosyl-(1->2)]-glucoside	1030.3995	23.9	$C_{65}H_{90}O_{10}$	-	527
131.	Unknown	1061.4968	28.2	-	-	275, 297, 311, 325, 343
132.	Unknown	1066.4534	28.3	-	-	545
133.	Unknown	1162.4263	22.9	-	-	593

Table S4: Table of identified highly expressed compound in most active extract of *T. cordifolia* co-occurring with *A. indica*.

**S. No.**	**Tentative Identification**	**Compound**	**RT**	**p (Corr)**	**AIN**
1.	Borapetosides D	698.427	27.4	7.39E-04	6.5
2.	N-Methylcoclaurine	299.164	24.4	6.49E-14	6.3
3.	Unknown	278.163	40.6	0.005105	3.6
4.	Isotanshinone II	294.230	30.2	0.048351	3.3
5.	Peonidin	301.309	38.1	5.93E-05	3.2
6.	5-Allyloxysalvigenin	714.264	17.8	1.35E-09	3.1
7.	Tinosporaside	500.169	18.5	4.34E-12	2.5
8.	Jatrorrhizine	338.144	28.8	3.71E-17	2.3
9.	Unknown	192.973	7.08	9.22E-10	2.2
10.	6-Methylcoumarin	160.058	23.9	3.46E-12	2.1
11.	Cinnamaldehyde	132.062	24.0	2.25E-11	2.1
12.	Unknown	317.304	34.8	3.61E-15	2.1
13.	Oblongine	313.179	25.6	2.99E-13	1.8
14.	3β,5α,14α-Trihydroxyergosta-7,22-dien-6-one	444.304	25.8	1.02E-16	1.8
15.	Cycloeucalenol	426.292	25.9	5.57E-16	1.8
16.	Trimethyldecahydrophenanthren-2-ol	248.114	25.8	1.30E-08	1.7
17.	20-Hydroxyecdysone	480.327	25.9	6.75E-14	1.7
18.	Chrysin	254.161	33.1	2.22E-08	1.7
19.	Unknown	273.277	34.7	3.18E-09	1.5
20.	Phenylmethanethiol	124.033	7.10	7.74E-15	1.4
21.	Cycloartane-24,25-diol-3-one	458.320	26.5	1.17E-11	1.4
22.	Palmitic amide	255.265	34.8	1.41E-10	1.4
23.	Palmarin	374.257	41.8	7.34E-06	1.4
24.	Betaine	117.084	6.77	1.72E-05	1.3
25.	Feruloyltyramine	313.143	25.4	1.84E-14	1.3
